# Toward a unified model of face and object recognition in the human visual system

**DOI:** 10.3389/fpsyg.2013.00497

**Published:** 2013-08-15

**Authors:** Guy Wallis

**Affiliations:** Centre for Sensorimotor Neuroscience, School of Human Movement Studies, University of QueenslandQLD, Australia

**Keywords:** face recognition, object recognition, learning and memory, holistic processing, neural network modeling

## Abstract

Our understanding of the mechanisms and neural substrates underlying visual recognition has made considerable progress over the past 30 years. During this period, accumulating evidence has led many scientists to conclude that objects and faces are recognised in fundamentally distinct ways, and in fundamentally distinct cortical areas. In the psychological literature, in particular, this dissociation has led to a palpable disconnect between theories of how we process and represent the two classes of object. This paper follows a trend in part of the recognition literature to try to reconcile what we know about these two forms of recognition by considering the effects of learning. Taking a widely accepted, self-organizing model of object recognition, this paper explains how such a system is affected by repeated exposure to specific stimulus classes. In so doing, it explains how many aspects of recognition generally regarded as unusual to faces (holistic processing, configural processing, sensitivity to inversion, the other-race effect, the prototype effect, etc.) are emergent properties of category-specific learning within such a system. Overall, the paper describes how a single model of recognition learning can and does produce the seemingly very different types of representation associated with faces and objects.

## Introduction

Our ability to recognize and analyze objects forms an essential part of our everyday life, and is something we achieve rapidly, accurately, and seemingly effortlessly. However, the apparent ease with which we accomplish this recognition is deceptive. This is perhaps nowhere more apparent than in the case of face recognition. Recognition across possible views of faces is hard, because faces change their shape as they rotate (profile, frontal view), they self-occlude (nose), they are non-rigid (expressions), they change over time (facial hair, aging), and very similar distractors exist (other faces). Understanding how humans achieve facial recognition is not only of interest to neuroscientists, but also to researchers from across the field of artificial vision, such as engineers involved in anything from robotics, border security, computer access, to camera phones. Given the task's complexity, one might think that scientists interested in unraveling the mysteries of visual processing in the human brain would do well to concentrate their efforts on more tractable issues first. However, in practice, visual recognition has proven a highly profitable model for the study of both visual processing and learning in humans because its goals are well defined. It has allowed scientists to probe both human and animal cortex in search of neurons which demonstrate the appropriate abstraction of visual information. Work of this kind has been central to the development of the two-stream hypothesis of vision (Ungerleider and Haxby, 1994), and has helped fuel debates about regional specialization in cortex, as well as the relative contributions of genetics and our environment to the behavior of neural systems.

Although outperformed by machines in some recognition tasks in recent years (O'Toole et al., 2007; Tan et al., 2013), our visual system appears particularly adept at discriminating, categorizing and identifying faces. On one level, this is perhaps understandable. Face recognition represents a potent drive to processes underlying natural selection, since it underpins appropriate interaction with the species most central to our survival, namely other humans (Öhman and Mineka, 2001; LeDoux, 2003). Whether it is in recognizing potential friendliness or threat from facial expressions; or identifying family, friends, clansmen or foes; correct performance is central to what the evolutionary biologists refer to as “fitness”. Whereas broad correct classification of animals, foods, tools and other objects might suffice for survival, correct within-category discrimination is essential for a functionally relevant face recognition system, since the behaviorally relevant question is often not “what is that?” (a face), but rather “who is that?”. From an evolutionary standpoint, then, faces may merit neural resources beyond those dedicated to other object classes. It turns out that there are numerous converging lines of evidence from developmental, neuropsychological (patient), behavioral and electrophysiological sources, that faces are indeed processed separately and/or differently to other objects, leading authors to argue that evolution has devoted specialist areas and pathways in the brain to the task of face recognition (Kanwisher et al., 1997; Öhman and Mineka, 2001; Tsao and Livingstone, 2008).

In this paper I discuss the evidence for face-specific processing from numerous sources, and attempt to clarify what results of this type tell us about the representation and recognition of faces. Using this preliminary review as a backdrop, I turn to evidence from some labs that many of the known effects are actually a symptom of expertise rather than something immutably unique to faces. I then go on to discuss a convergence in thinking that exists between scientists working in the traditionally isolated domains of face and object recognition, arguing that the main missing ingredient has been a consideration of the effects of learning. I argue that by turning to a more biologically relevant, self-organizing, competitive system (one which allows the visual diet of the observer to shape the classifiers that are formed), classic face-like properties such as holistic processing spontaneously emerge as a function of visual experience.

Ultimately, the self-organizing model described here helps explain how the many undeniable peculiarities of face recognition represent emergent properties of a standard model of object recognition in which a small subset of stimuli are highly over-trained.

## The special properties of faces

There has long been a debate as to the “specialness” of faces compared to other objects (Ellis and Young, 1989; Gauthier and Logothetis, 2000; Bukach et al., 2006; McKone et al., 2007). But for many working in the area, there remains little doubt that faces are special in a number of ways, and that the debate is hence more-or-less at an end (McKone et al., 2007). This is not simply a view held by those working in the domain of face recognition. Some of the world's most senior theoreticians working in the area of object recognition have argued that the processing of faces is unlike that of other objects (Biederman, 1987; Biederman and Kalocsai, 1997; Leibo et al., 2011).

One significant aspect of face processing often discussed is the apparently holistic manner in which faces are processed (Tanaka and Farah, 1993; Carey and Diamond, 1994; Schwarzer, 1997; Farah et al., 1998; Peterson and Rhodes, 2003). Support for a holistic model comes from a number of sources: First, jumbling nameable parts (mouth, nose, eyes) leads to reductions in both recognition speed and accuracy (Tanaka and Farah, 1993; Farah et al., 1998). Second, discrimination based upon the upper half of the head, say, is disrupted by the presence of the lower half of another person's head when the two halves are aligned, suggesting an inability to process the two halves independently (a result termed the “composite effect”) (Young et al., 1987; Hole, 1994). Although there is some evidence that other objects of expertise also reveal a composite effect, the studies remain controversial (Rossion, 2013).

As well as being sensitive to the conjunction of nameable parts, human observers are also sensitive to placement of those parts within a face (Leder and Bruce, 1998; Maurer et al., 2002). Any slight change in the distance between the eyes or between nose and mouth etc. can greatly affect recognition performance (termed the “configural effect”). Studies of this effect have tended to argue that this is because configuration in and of itself matters (Maurer et al., 2002). However, this interpretation has been challenged on technical grounds (McKone et al., 2007), and more carefully controlled experiments have produced very different results (Riesenhuber et al., 2004; Sekuler et al., 2004; Yovel and Duchaine, 2006). Where configural effects have been demonstrated it may be safer to interpret them as evidence that humans are sensitive to the configuration of nameable facial parts—i.e., further evidence for holistic processing. Two very recent studies which further corroborate the idea of cortically localized holistic processing, come from patients subjected to cortical stimulation. Both described periods of breakdown in the facial whole in which features appear in the wrong places within the face, an effect which rapidly ceased as soon as stimulation stopped (Jonas et al., 2012; Parvizi et al., 2012).

Face recognition also generalizes very poorly across planar rotation i.e., turning the face upside down (termed the “inversion effect”) (Yin, 1969)^1^. In the past, some have claimed that the inversion effect is due to a complete breakdown in holistic processing when faces are inverted (Thompson, 1980; Leder and Bruce, 1998; Maurer et al., 2002). However, more recent studies have argued that the full story is unlikely to be that simple (Valentine and Bruce, 1985; Sekuler et al., 2004; Talati et al., 2010).

There are certainly many other aspects of face processing which are unusual, including developmental studies in babies (based on preferential looking); the face-specific recognition deficit prosopagnosia (Behrmann et al., 2005; Duchaine et al., 2006; Yovel and Duchaine, 2006); the face-selective centers of the brain (Fusiform-face area or FFA, see later), enhanced processing of certain facial expressions (Öhman and Mineka, 2001; Horstmann, 2007) [For a critical review and new data see Coelho et al. (2010) and Calvo and Nummenmaa (2008)]. There are also electrophysiological effects unique to faces. For example, there is evidence for pronounced electrical activity associated with seeing faces (called the N170, see Thierry et al. (2007) and Boehm et al. (2011) for a critical review and new data). I will say more about some of these effects in the coming sections, but will restrict discussion to studies which speak directly to how cortical representations are established and what form these representations take.

## The representation of faces and objects in temporal lobe cortex

Current understanding of the primate visual system points to the fact that the task of both face and object recognition is centered on a pathway leading from primary visual cortex, in the occipital lobe, down into the inferior (lower) sections of the temporal lobe (Ungerleider and Haxby, 1994; Logothetis and Sheinberg, 1996). Consistent with this hypothesis, damage to temporal lobe cortex can lead to specific recognition deficits such as the associative agnosias described in patient studies (Farah, 1990). This in turn mirrors recording in the homologous region of monkeys which has identified cells responsive to faces and other familiar objects (Desimone, 1991; Rolls, 1992; Logothetis and Pauls, 1995; Baker et al., 2002). Brain imaging studies in healthy humans have likewise revealed selective activation of temporal lobe areas during recognition tasks involving faces and other objects (Kanwisher et al., 1997; Gauthier et al., 2000; Haxby et al., 2001). Recent work looking at the single cell responses of humans in special patient groups have served to further reenforce this picture (Quiroga et al., 2009). The electrophysiological studies in particular, have revealed that the further one looks along the object recognition pathway, the larger the spatial extent over which individual neurons respond, and the greater the tolerance they exhibit to changes in an object's location and size (Desimone, 1991; Rolls, 1992; Perrett and Oram, 1993). On the basis of receptive field sizes and neural response times, it appears that true view invariance comes last of all, at a stage in which size- and location-tolerant neurons are pooled to form view-invariant responses (Perrett et al., 1987, 1992; Logothetis and Sheinberg, 1996).

There have been a great deal of studies conducted looking at the selectivity of temporal lobe neurons. Perhaps the best source of information currently available comes from single cell recording and optical imaging studies in the macaque, as well as recent single unit studies in humans. This work has revealed cells which can be effectively stimulated by sub-parts of a full object often irrespective of precise size or location (Yamane et al., 1988; Tanaka et al., 1991; Tsunoda et al., 2001). In a particularly revealing study based on intrinsic imaging, Wang et al. (1996) describe groups of neurons equally responsive to a feature (e.g., the silhouette of a cat's head) or any object containing that feature (cat); whereas other neural centers appeared more integrative/holistic (only the whole cat was an effective stimulus). There have been numerous other reports of highly selective sensitivity in temporal lobe neurons (Desimone, 1991; Tanaka et al., 1991; Logothetis and Pauls, 1995).

Despite the undeniably high levels of stimulus selectivity, studies of within-category selectivity of face cells suggest that even neurons from the most anterior parts of the temporal lobe respond to many of the faces tested (Perrett et al., 1992; Young and Yamane, 1992; Abbott et al., 1996). Scientists recording a decade later made the same informal observation: “Although some cells responded best to only one or a few faces, many cells were responsive to a wide variety of face images, including familiar and unfamiliar faces, human and macaque faces, and even cartoon faces” (Tsao et al., 2006). Hence the overall conclusion appears to be that cells in this region can be highly selective for a specific set of stimuli, but that they rarely respond to a single stimulus, indicating that the representation in this area falls short of becoming completely holistic. Instead, the neurons appear to be sensitive to specific pictorial subregions or broad shape cues such as the outline of a head. Some neurons do appear selective for nameable parts (as predicted by Tanaka and Farah, 1993), but this appears to be the exception rather than the rule.

The early studies in monkeys generally reported an inter-mingled pattern of cell selectivity, with the relative density of face cells peaking at around 20% (e.g., Perrett et al., 1982). Later studies by Tanaka et al. (1991) tackled the task of characterizing responses of the other 80% of cells. The group went on to describe the orderly clustering of these cells in terms of their preferred visual stimuli, while at the same time highlighting the rich intermingling of these clusters (Fujita et al., 1992). It is worth bearing in mind that this picture of inter-mingled neural selectivity was based on cytoarchitectonic (anatomical) regions. More recent work by Tsao et al. (Tsao et al., 2006, 2008b; Moeller et al., 2008; Freiwald and Tsao, 2010) chose to define regions of interest functionally, using fMRI. They reported very high concentrations of face-selective cells, as well as interconnected, face-selective “patches” running through occipital and ventral cortex (see also Zangenehpour and Chaudhuri, 2005). As well as appearing to link up more closely with the phenomena of prosopagnosia, Tsao and colleagues' work accords with functional imaging work in humans which has repeatedly singled out a sub-region of inferior temporal lobe (called the fusiform face area or “FFA”) as being strongly activated by faces (Sergent et al., 1992; Puce et al., 1995; Kanwisher et al., 1997). Beyond faces, there is growing evidence for regional specialization of function in temporal cortex for other visually acquired objects such as written words (McCandliss, 2003; Glezer et al., 2009; Pegado et al., 2011) and in tool use (Mahon et al., 2007) amongst others.

## Evidence for learning in visual recognition

Although many aspects of face recognition have been carefully characterized and we now know a great deal about the types of cells that support recognition, the means by which they are established remains a matter of debate. This section lays out the evidence for learning by combining evidence from behavioral, theoretical and electrophysiological sources.

### Background

At the cellular level there is little doubt that temporal lobe neurons represent a significant substrate for learning in visual recognition. Rolls et al. (1989), for example, were able to demonstrate rapid adaptation of a neuron's selectivity for faces. In addition, both Miyashita (1988) and Kobatake et al. (1998) found cells in the temporal lobe responsive to artificial stimuli used in previous training, a fact which could not easily be explained by natural biases or innate selectivity. Kobatake et al. (1998), in particular, demonstrated that the number of cells selective for a trained stimulus was significantly higher in a trained monkey than in the cortex of naive monkeys and Baker et al. (2002) demonstrated that the neural representations of novel objects become more specific and integrated with training. Logothetis and Pauls (1995) trained monkeys to recognize particular aspects of a novel object class (see Bülthoff and Edelman, 1992). After training, many neurons were shown to have learned representations of particular objects including some neurons that were selective to specific views.

Learning in temporal lobe cells can be built up over many months, but can also be almost instantaneous, reflecting behavioral changes measured in human responses to stimuli. Tovee et al. (1996), for example, presented camouflaged, two-tone images of faces (“Mooney Faces”) to monkeys. Some neurons which did not respond to any of the two-tone faces did so if once exposed to the standard gray-level version of the face. This accords with findings in humans, who often struggle to interpret two-tone images at first, but then have no difficulty interpreting the same image even weeks later.

Apart from the evidence for the experience-dependent modification of neural responses, there are also ample examples from behavioral studies of face and object recognition. One important development in the last years of the 1990's was the introduction of stimuli chosen from novel object classes. What emerged from this work was that if two views of a novel object were learned, recognition was better for new views oriented between the two training views, than for views lying outside them (Bülthoff and Edelman, 1992; Edelman and Bülthoff, 1992). More recently, studies based on functional imaging data have reported large-scale changes to the organization and selectivity of temporal lobe cortex in humans after training. They have also highlighted how the changes are related not only to the stimuli used but also the recognition task involved (Op de Beeck et al., 2006; Gillebert et al., 2009; Wong et al., 2009b).

Although many models of object recognition deny (Olshausen et al., 1993) or are indifferent to the precise mechanisms of learning (Fukushima, 1980; Riesenhuber and Poggio, 1999), one group of models predicts that all forms of tolerance to changes in appearance are learnt (Földiák, 1991; Wallis, 1998; Wallis and Bülthoff, 1999). Behavioral evidence to support the hypothesis came originally from face recognition studies. The studies looked at depth rotation (Wallis and Bülthoff, 2001; Wallis, 2002) and later planar rotation and illumination changes (Wallis et al., 2009), but related work has revealed parallels with non-face stimuli too (Stone, 1998; Vuong and Tarr, 2004; Liu, 2007). DiCarlo et al. have also made progress discovering the neural substrates of such learning in macaques, with reference to location and size invariance learning (Cox and DiCarlo, 2008; Li and DiCarlo, 2008, 2010). Related effects have also recently been reported in a study of spike dependent plasticity (McMahon and Leopold, 2012). The fact that both the face and object recognition systems are amenable to the same type of learning does not, of course, necessarily imply that they are subserved by the same system, but it does suggest that if separate systems exist, they are subject to similar mechanisms of learning. Certainly, work on other functionally defined areas in the temporal lobe, such as the Visual Word Form Area (McCandliss, 2003; Cohena and Dehaene, 2004), strongly suggest that regions of specialization can emerge for “non-prepared” (i.e., manmade) stimuli, opening the possibility that face specific regions emerge through experience too.

### The issue of expertise

One of the most hard-fought, sometimes rancorous debates in the field of object and face recognition literature, concerns the role of learning in face recognition, and in turn the issue of visual expertise. Few would disagree that there are regions of cortex filled with face-selective neurons, or that the neurons supporting recognition learn from experience. Where agreement breaks down is on the issue of how these representations are established and why. Many researchers have taken the selectivity of FFA as evidence for a face-specific system dedicated to the task of face processing (Kanwisher et al., 1997; McKone et al., 2007; Liu et al., 2010), whilst others have argued that there is no specialist region for face processing *per se*. Instead, faces are seen an example of an object category in which most of us are experts and that FFA is selective to any and all objects of expertise (Gauthier and Tarr, 2002; Bukach et al., 2006; Gauthier et al., 2009).

Although this might appear to be a debate which would lend itself to empirical test, the truth is that arguments about experimental methods and the interpretation or reliability of specific results have allowed the debate to rumble on. One early source of evidence for the expertise hypothesis came from Diamond and Carey (1986) who described the high sensitivity of dog experts to picture-plane inversion compared to control subjects, suggesting face-like sensitivity to a non-face category of expertise. However, a recent study by Robbins and McKone (2007) has cast doubt over those results after they failed to replicate the effects. The follow-up debate to their article is worth reading because it highlights numerous areas of disagreement between representatives of the two sides of the debate (Gauthier and Bukach, 2007; McKone and Robbins, 2007). One criticism which the Robins and McKone study has to tackle is the fact that their dog experts performed relatively poorly at the tasks they were set, relative to young naive volunteers. The authors argue that one should look to the worse performance of age-matched controls. Nonetheless, as the Busey and Vanderkolk (2005) study of fingerprint experts shows, it is possible for experts to outperform all-comers of all ages (even academic trained, younger volunteers). It would be interesting to find a task that the dog experts were truly good at. One candidate task, mentioned in passing by the authors, might be the experts' ability to correctly guess the country of origin of the dogs.

On a broader level, what the debate about expertise reveals is that it can be hard to devise the right stimuli and tasks to conduct meaningful human behavioral testing. This was a problem which hampered the object recognition debate for many years. Those that argued for view-independent representations pointed to results using between-category performance on familiar objects, and those that advocated a view-sensitive representation pointed to results from studies using within-category discrimination of novel object classes (Biederman, 1987; Bülthoff and Edelman, 1992; Biederman and Gerhardstein, 1993; Tarr and Bulthoff, 1998). In the case of faces, one can look at the results of Duchaine et al. (2006) on prosopagnosia. Their results reveal that for a particular level of task difficulty a prosopagnosic may appear to show relatively normal face discrimination ability, perhaps based on local, diagnostic features (large eyes, distinctive nose). Nonetheless, with appropriate controls and changes to noise levels or view point, the prosopagnosic's approach to face discrimination fails, and performance rapidly drops off.

One important lesson to emerge from the debate on object recognition was that in order to understand the current system and its abilities it can be advantageous to take a stimulus set which is completely novel, so as to permit monitoring of the development of tolerance to changes in appearance over time. This approach was adopted by Gauthier et al. in attempting to understand the possible role of expertise in face recognition. They created numerous novel stimulus sets including “Greebles” (nonsense creatures made from simple geometric parts). Their studies revealed how repeated exposure to these novel stimuli gradually yielded sensitivity in their observers to image properties normally regarded as specific to face processing, including configural and composite effects (Gauthier and Tarr, 1997, 2002; Ashworth et al., 2008). There is a wealth of behavioral evidence to support the idea that holistic processing emerges only after high levels of exposure, both in the object and developmental face recognition literature. For a recent and extensive review of that evidence one can turn to Crookes and McKone (2009), who then go on to explain why they believe the majority of the results are unreliable because of a failure to match task difficulty across the different age ranges. Their work is not uncontroversial but it does, once again, highlight the difficulties associated with choosing appropriate stimuli and tasks for behavioral experiments.

A significant element of the expertise story has focussed on the specificity of FFA. In a series of papers Gauthier et al. demonstrated that the FFA of subjects also responded to objects of expertise including an artificial object class (Gauthier and Tarr, 1997), and real-world object categories such as cars and birds (Gauthier et al., 2000). A later study questioned whether FFA was necessary for face categorization (Haxby et al., 2001), and high resolution analysis of FFA indicates that the classically defined FFA is actually selective to things other than just faces (Grill-Spector et al., 2006). At the same time it would be fair to say that the results of some of these earlier studies have been subjected to close scrutiny, resulting in a partial retraction in one instance (Grill-Spector et al., 2007). Also, new experiments have suggested that it was actually facial elements of Gauthier and other's “stimuli of expertise” which were responsible for activating FFA (Brants et al., 2011). But the idea has certainly not disappeared (Gauthier et al., 2009) as some might have wished (McKone and Robbins, 2007). Indeed, recent studies employing high field fMRI with 1 mm^3^ voxels, have again argued that FFA is linked to expertise (McGugin et al., 2012) or at least contains multiple centers responsive to multiple stimulus types (Weiner and Grill-Spector, 2012). Also, attempts to decode the representation in FFA suggest that anterior IT may contain more useable information for face discrimination (who is that?) than FFA, which was more attuned to the task of categorization (face vs. non-face) (Kriegeskorte et al., 2007).

Whatever the precise role of FFA in face processing, as Crookes and McKone (2009) themselves point out, one fact in favor of the expertise hypothesis in that the size of FFA increases substantially throughout childhood and into early adulthood (Golarai et al., 2007; Scherf et al., 2007). Apart from suggesting an exposure driven model of cortical specialization, it also suggests that the face and non-face specific areas are not so functionally distinct as some compartmentalized models of temporal lobe selectivity might suggest, since recruitment of non-face specific areas for face selective activities is possible.

As mentioned in passing earlier, work on visually evoked potentials (using EEG equipment) has provided evidence that faces produce an enhanced negative potential at around 170 ms post stimulus onset (Bentin et al., 1996). Of relevance to the debate on expertise, a study of experts in fingerprint analysis revealed a delay in their N170 responses to inverted fingerprints which was not present in control subjects, apparently mirroring the delay found for faces (Busey and Vanderkolk, 2005). It should be added that the meaning of the N170 is a matter of forceful, ongoing debate (Thierry et al., 2007; Rossion and Jacques, 2008), but that debate is centered on the difficulty of comparing stimulus responses across stimulus sets as heterogeneous as cars, houses and faces. In the case of the Busey and Vanderkolk (2005) study, the comparison is based on the same (fingerprint) stimuli, making the difference all the more striking.

One of the best pieces of evidence for learning in the face recognition system is the “other-race” effect (Chance et al., 1982). This refers to the fact that observers are faster and more accurate at discriminating faces from their own race than those belonging to an unfamiliar race. On the basis of this single piece of evidence alone, it seems that some aspects of face recognition must be affected by levels of visual exposure and hence expertise. Researchers have speculated in the past that our inability to discriminate faces of races other than our own might be related to a lack of holistic coding of other-race faces (Rhodes et al., 1989), a proposal which has received recent empirical support (Michel et al., 2006; Rossion and Michel, 2011). The plasticity of these effects has been further enforced by reports of an “own-age” effect, in which discrimination performance is biassed toward the age-range of ones peers (Hills and Lewis, 2011; Hills, 2012).

## Models of visual recognition

### Background

Having reviewed what is “special” about faces and what is known about the neural basis of face and object recognition, it is time to turn to more formal models of how faces and objects are represented, and how these representations are established. Models from the two fields of object and face recognition have evolved largely independently of one another but in this section I will describe reasons for thinking that models in the two fields are in fact intimately related.

We can begin the section by asking a question: How would a self-organizing recognition system respond to seeing huge numbers of a single class of objects? One can test this easily enough theoretically, but in order to seek parallels behaviorally, one would have to ask volunteers to look at a specific stimulus for hours a day over a period of weeks. To really test a system one might add the constraint that participants could only look at upright versions of those stimuli. Only then could one begin to truly assess the impact of this type of biassed sampling of the input space. The only issue is, who would want to do an experiment of this type? It turns out, of course, that the experiment I am describing exactly parallels our daily experiences with faces. Couched in these terms, face recognition suddenly feels like a rare opportunity to test object recognition theories to destruction. In the following sections I will attempt to describe how over-learning of a specific class of stimuli causes self-organizing systems to produce peculiarly specialized feature analysers. The analysers are more holistic than is the case for analysers focussed on other everyday objects, with the result that a sub-system emerges with relatively high sensitivity to change (good discrimination performance) but also relatively poor generalization, especially across novel transformations (such as inversion).

### Objects

Classical approaches to object recognition have focussed on deconstructing the retinal image into cues relating to 3D shape such as depth and edge junctions (Marr and Hildreth, 1980; Biederman, 1987). Other models posit the presence of neural circuitry for conducting transforms of size and location on arbitrary forms (Graf, 2006), while others argue for the existence of object prototypes (Edelman, 1995). An alternative model proposes that recognition is based upon image matching (Poggio and Edelman, 1990; Bülthoff and Edelman, 1992) and more recently, abstract feature matching (Wallis and Bülthoff, 1999; Ullman, 2006; Torralba et al., 2007). In its simplest form, the image-based approach can be thought of as representing objects through a series of snap-shots taken under varying viewing conditions (lighting, viewing direction, size, location etc.). Recognition simply requires matching new images to any one of the stored images. By switching to features, rather than whole views, experience with one object can transfer immediately to other objects, allowing novel objects to be recognized from untrained viewpoints (see Wallis and Bülthoff, 1999).

Despite its ability to transfer experience to other views and objects, one important aspect of the feature-based model is that it predicts imperfect generalization across view changes. This actually accords perfectly well with a host of behavioral data on faces and novel objects. For example, humans are less than perfect at generalizing across depth rotations or across extreme lighting conditions (Patterson and Baddeley, 1977), and many aspects of object recognition are not truly transform invariant for novel object classes without training (see Edelman and Bülthoff, 1992; Graf, 2006). This need for learning also accords with what we know about face and body selective neurons in the temporal lobe which do not natively generalize recognition across all object sizes and locations (e.g., Ashbridge et al., 2000).

As well as its appeal in terms of biological plausibility, the feature-based model has been shown to have explanatory power for a number of well known behavioral phenomena in the field of object recognition. For example, it has long been known that the time required to recognize an object from a new viewpoint correlates with the view's disparity from a previously learned view (Shepard and Cooper, 1982). Many have interpreted this as evidence for the presence of a rotatable, internal 3-D model. However, it turns out that such effects are also predicted by a distributed, view-based representation (Perrett et al., 1998).

Despite the improvement in generalization which a feature-based approach brings over the strictly view-based one, a significant problem that these models faced in the past was to explain how to associate very different looking views of a single object into a unified representation. Many models side-step the issue by using supervised learning schemes (Poggio and Edelman, 1990; Riesenhuber and Poggio, 1999). This is a problem that requires solving however. A standard, self-organizing (e.g., Hebbian) system associates on the basis of physical appearance. Associating object views according to physical similarity can, at best, only provide limited tolerance to variations in an object's appearance (a head can look quite different when seen from different directions). A plausible and robust solution appears to be that the visual system associates views on the basis of their temporal proximity as well as spatial similarity (Pitts and McCulloch, 1947; Földiák, 1991; Miyashita, 1993; Wallis and Bülthoff, 1999, Wallis et al., 2009). Temporal proximity is informative because images streaming into our visual system are likely to be views belonging to a single (possibly transforming) object. As we turn a box in our hand, for example, it produces a stream of reproducible, temporally correlated views. Associating views in this way has the advantage that it is useful for invariance learning across all manner of naturally occurring transformations including rotation in depth, spatial shifts and in-plane rotations, size changes, illumination changes, non-rigid motion, and so on. Temporal association appears to offer the missing ingredient for a system that can operate and organize fully autonomously, being guided by the statistical regularity in time as well as space of the input it receives. Network simulations have demonstrated how a minor modification to standard Hebbian association (called the trace rule) can produce view change tolerant representations in self-organizing systems (Földiák, 1991; Becker, 1993; Wallis et al., 1993; Wallis, 1998)—see Rolls (2012) and Bart and Hegdé (2012) for recent reviews. Subsequent electrophysiological studies have leant further support to this theory (Cox et al., 2005; Cox and DiCarlo, 2008; Li and DiCarlo, 2008, 2010) which has prompted developers of other hierarchical models of object recognition to experiment successfully with trace-rule learning (Isik et al., 2012).

### Faces

Despite the widespread use of feature-based models in object recognition, it is apparent that their users have rarely had anything specific to say about face recognition. Most of the theoretical work on face processing has proceeded independently of progress in the field of object recognition. Within the face literature, debate has largely centered on norm-based, prototype, exemplar-based, or configural models (Valentine, 1991; Maurer et al., 2002; Rhodes and Jeffery, 2006). For many working in the area, evidence points to a norm-based model in which faces are encoded relative to the central tendency of faces we are familiar with (see e.g., Leopold et al., 2006; Rhodes and Jeffery, 2006; Susilo et al., 2010), but as Valentine (1991) pointed out, both exemplar and norm-based models can account for a whole range of behavioral phenomena including the other-race effect and the independence of distinctiveness and familiarity. In the end he offered this telling insight: “...difficulty in discriminating between the [norm-based and exemplar-based] models arises because exemplar density is assumed to be correlated to distance from the norm.” Crucially, what I assume he means here is that the density of exemplars decreases with distance from the mean, i.e., density is *inversely* correlated with distance, which in turn means the density of classifiers also goes down, leading to a natural decrease in sensitivity to changes in facial appearance (see Davidenko and Ramscar, 2006). In a subsequent paper in which Valentine directly manipulated distinctiveness within the context of the other-race effect, he felt able to conclude that the exemplar-based model offered a more parsimonious explanation for the effects than a norm-based one (Valentine and Endo, 1992; Valentine, 2001).

In practice, exemplar-based models like Valentine's fell out of favor in the face-recognition community for some years because they appeared unable to explain the advantage afforded by caricatures to recognition performance, something a norm-based model is well placed to explain. However, later developments of exemplar models have successfully tackled these issues. Only a few years after the release of Valentine's seminal papers, a study simultaneously manipulating race and caricatures and concluded that an exemplar-based model better explained the interactions measured (Byatt and Rhodes, 1998). A year later (Lewis and Johnston, 1999) described an elegant reworking of the exemplar idea based on an explicit connectionist model. While some details were not addressed, such as the exact neural basis of the representations or how the representations are established, the strengths and consequences of an exemplar-based representation were now clearly conveyed. Their results and simulations dovetail nicely with work in my own lab on the prototype effect (Wallis et al., 2008). In that paper, my colleagues and I reported evidence for an abstract featured-based (multi-channel) model of face recognition based on self-organizing principles which, despite being derived from a model of object recognition, bears close analogy to the face-space classifiers which (Lewis and Johnston, 1999) describe. I have more to say about the caricature effect in the Appendix section of this paper.

Like Valentine before him, Lewis and Johnston took their results as evidence for an exemplar-based model of face representation. For those supporting the norm-based model, there remains significant evidence that exemplar-based models are inadequate, because they cannot explain the face adaptation after-effect (Leopold et al., 2006; Rhodes and Jeffery, 2006; Susilo et al., 2010). Although beyond the scope of this paper to fully review, there are multi-channel models which can account for this effect too if one assumes that although adaptation is happening to the multi-channel features, adaptation effects are filtered through a subsequent, binary decision process (e.g., Ross et al., 2013).

Nonetheless, from the perspective of those working on face recognition, the feature-based model simply cannot account for several important behavioral effects. For example, because observers are sensitive to the spacing between nameable parts (eyes, nose, mouth, hairline etc.), some theorists have concluded that we must represent faces using a code based on facial metrics, i.e., distances between facial landmarks such as the eyes, tip of the nose etc. (Leder and Bruce, 1998; Maurer et al., 2002). Although evidence for such a model has waned, the configural and composite effects still seems to speak against recognition based on localized facial features. The crucial point to bear in mind, however, is that the features being described here are not simply nameable features. They are *abstract*, meaning they can span nameable parts and will vary in physical extent across the face. We know that some neurons respond to large-scale properties such as head shape, for example, whereas others respond to something as specific as a mouth with appropriate texture and color properties (Rolls, 1992; Tanaka and Farah, 1993). At the same time, abstract features are not simple 2D templates in that they often maintain their response across changes in viewpoint, location and size. Overall, they are tuned to elements of a face in such a way that they might respond to as many as 10% of all faces tested (Rolls, 1992; Wallis and Bülthoff, 1999).

In the end, a closer inspection of the literature does find examples of the use of exemplar-based models to explain face recognition. Valentin et al. (1997), for example, explained how a distributed, view-based system predicts the 3/4-view pose recognition advantage for faces despite the predominance of cells selective for front and profile views (Perrett et al., 1987). The same team has offered experience- plus feature-based accounts for the other-race effect as well, as I will describe later. Furthermore, Brunelli and Poggio (1993) explicitly tested feature-based vs. configuration-based classification for faces and found that their feature-based algorithms consistently outperformed metrics-based ones. In a more recent and more explicit attempt to bridge the face-object divide, Jiang et al. (2006) showed how a feature-based, biologically inspired model of object recognition is capable of mimicking a number of aspects of face processing including the inversion and configural effects. In practice, through, their approach involved fitting model parameters to the desired selectivity and hence it can be seen as a proof of concept, but falls short of explaining how and why encoding takes on this form for faces and not other objects.

### Unifying models of face and object recognition

So how might all of these strands be drawn together to form a viable model of both object and face recognition? A useful starting point is to consider how current models of object recognition work. Inspired by the known hierarchical organization of visual cortical areas (Rolls, 1992), many biologically relevant models of object recognition incorporate a convergent hierarchy of neurons organized into layers (Fukushima, 1980; Wallis and Rolls, 1997; Riesenhuber and Poggio, 1999). Although initially restricted to toy problems, recent simulations using this family of models have demonstrated how well the system scales up to tasks that come close to real-world scene analysis (Serre et al., 2007).

Irrespective of the precise implementation, one of several design aspects which these models have in common is the idea that each layer contains pools of mutually inhibitory neurons, each striving to fire most strongly in response to a stimulus and to actively suppress firing in neighboring neurons. If it is not immediately clear why a neuron should want to maximize its firing, not least in light of theories of coding based on sparseness or efficiency (e.g., Baddeley et al., 1997), it is perhaps worth reflecting on the impact of Hebbian association, characterized by the phrase “fire together, wire together”. Hebbian association requires a neuron to tune its input weights in such a way as to enhance its response to inputs that caused it to fire in the past, hence it is driven to respond more effectively and efficiently assuming inputs repeat over some reasonable time interval. What constrains them from firing all the time is the inhibitory input they receive from their neighbors, and some presumed limited resource of synaptic weight which has to be shared across their synapses (Hertz et al., 1990).

Neurons satisfy their desire to be active by employing a mixture of two strategies: (1) A neuron focuses in on a narrow region of the input space in which only a few exemplars exist, but these exemplars are seen relatively often. Despite the limited number of stimuli which it can respond to, it is activated relatively often because those few stimuli occur frequently. (2) A neuron may choose to be less selective, responding to a broad range of stimuli which occur only occasionally. Although each of its preferred stimuli appear relatively infrequently, the neuron fires regularly because any one of a wide range of these occasional stimuli will activate it. The choice of which strategy to employ is not the neuron's to make of course, but is instead governed by three factors: (1) the statistical properties of the input it sees; (2) the neuron's initial selectivity; and, critically, (3) the selectivity of neurons responding to neighboring regions of the input space.

**Figure 1:**
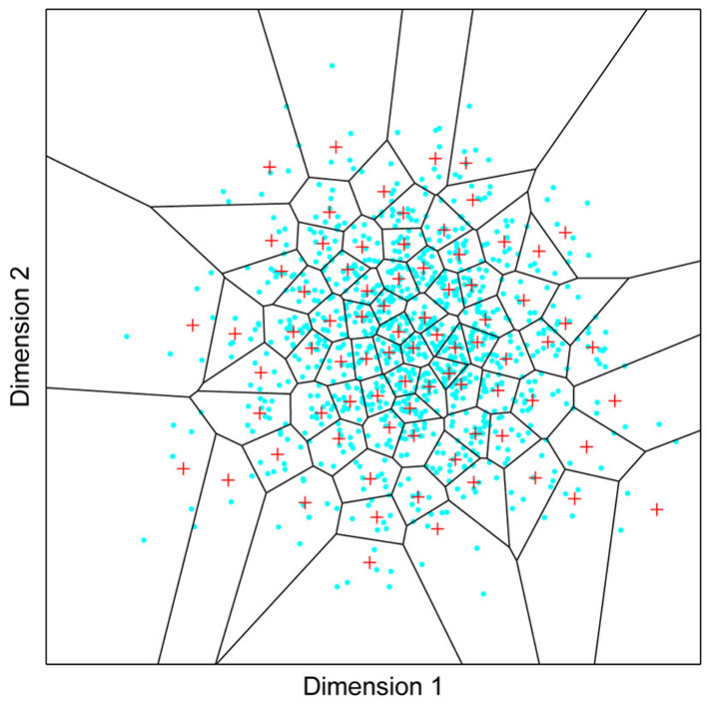
**A model of face space in which the physical appearance of a set of 1000 faces is assumed to vary normally along two feature dimensions.** Each “Dimension” could correspond to nameable features such as nose width, hair color, eye separation etc., but they might just as well be complex combinations thereof and hence be hard to characterize in words. The red “+” points represent the distribution of neural classifiers which emerge from a self-organizing clustering algorithm exposed to the faces. In other words the “+” markers indicate the neural weight vector of neurons which serve as the set of abstract-feature classifiers. Light blue dots represent locations of the individual faces and the black lines indicate the boundaries of face space within which the enclosed neural classifier fires most strongly—making this a Voronoi diagram similar to that used by Lewis and Johnston (1999).

In order to understand how face processing would proceed in a competitive system, it is important to reflect on the effect of regular exposure to a particular object class, i.e., the development of expertise. Stimuli falling within an area of expertise are seen very often. This makes the associated feature inputs a prime target for neurons within a competitive system. A neuron will adapt its input selectivity so as to maximize its response to an input corresponding to an oft repeated feature of that object class. However, it is not alone. The sphere of interest of other neurons will also tend to migrate toward the epicenter of input activity. In the end, the relatively high density of inputs in a region of expertise draws in large numbers of neurons and the resulting competition with very similarly tuned neurons, drives these “expert” neurons to integrate ever more aspects/features of their favored stimuli. As a result of competition, these neurons start to develop selectivity to information from across multiple dimensions/features of the stimulus, resulting in a more holistic representation. In contrast, neurons focussed on regions of the input space containing objects which are seen less frequently, experience less crowding from neighboring neurons. They remain relatively unselective across many dimensions of the input space, perhaps focussing on a single diagnostic feature. To illustrate this point see Figure 1 which captures these ideas based on a hypothetical competitive system exposed to inputs characterized by two feature dimensions.

For many readers the representation in the figure should be relatively familiar. But for those of you less accustomed to looking at such things, it is important that the concepts are made clear as this type of representation will form the basis for the first model described in this paper. In the figure, the two axes, labeled “Dimensions”, represent two physical dimensions along which faces can be represented. They might correspond to something tangible, such as aspects of a person's mouth or nose, but in practice they are likely to be more obscure combinations of multiple properties of a face. Nonetheless, for sake of illustration let us assume that they do indeed correspond roughly to the size of two nameable parts: nose length (Dimension 1) and mouth width (Dimension 2). In the figure, each of the light blue dots can be thought of as a face which an observer has seen at some point in her everyday life. Each person she encounters has a particular length of nose and width of mouth, and these properties correspond to a position in the two-dimensional space portrayed in Figure 1. The red crosses represent the corresponding location of a neural weight vector overlayed on the same pair of input dimensions. One can think of it as a representation of the optimal nose length and mouth width for producing the strongest activation of the neuron in question. As we can see, some neurons learn to respond strongly to faces bearing short noses and wide mouths, whereas others respond well to wide mouths and big noses, etc. The black lines represent the boundaries within which each neuron “wins”. Any face corresponding to a location within the region demarcated around a neuron's weight vector (“+”) will cause that neuron to fire the strongest of all.

Note that in this analogy, if a face appears that has a long nose and narrow mouth (bottom right corner of the input space), there are relatively few neurons covering the corresponding region. The result is that minor changes to that face will not produce noticeable changes in the neural response, because the same (broadly tuned) neuron is likely to fire strongly to both versions of the face. This implies poor discrimination of stimuli falling in this region of input space. By contrast, a face falling in the middle of Dimensions 1 and 2 will sit within a highly clustered zone with lots of neurons vying to respond to it. Any minor changes in the input are likely to be reflected in significant changes in the neural response of the system because the face is likely to move from the “win zone” of one neuron into another. In other words discrimination performance for faces falling into this zone will be high.

In many respects the selectivity of the neurons echoes the distribution of the faces in the input space, and hence echoes Valentine's description of face space and all of the associated emergent properties which it brings (see later). For the moment is sufficient to realize that zones of face-space containing lots of faces generate lots of narrowly tuned neurons all tightly packed together, whereas zones with fewer examples contain correspondingly fewer, more broadly tuned neurons. As Jiang et al. (2006) describe, a multi-channel model is capable of producing configural and inversion effects as long as the neurons are tightly tuned to the input stimuli. High exposure to upright faces is likely to produce just this type of representation in face-sensitive neurons due to the “crush” of many neurons focussed on the relatively small region of the visual input space occupied by faces.

## A hebbian model of face space

### Introduction

As described in the introduction, the aim of this paper is to investigate whether the architectural and functional bases of object and face recognition can be regarded as being fundamentally the same. To make this more concrete, this section combines models of human object recognition (Wallis and Rolls, 1997; Riesenhuber and Poggio, 1999) with the face space exemplar approach of Valentine (1991) and its supervised neural implementation (Lewis and Johnston, 1999). The resultant model is then used to explain behavioral phenomena specifically associated with face processing.

The model is kept deliberately simple because in many ways, the message is simple: Any self-organizing (unsupervised) competitive system will produce holistic (high-dimensional) representations of their preferred stimuli if the neurons are tuned for stimuli which fall in an area of high exemplar exposure (an area of expertise). There are many more sophisticated models of object recognition which one could consider, but the simplicity of this model is intended to demonstrate the generality of the proposal that holistic processing, expertise, competitive neural processes, and learning are intrinsically linked.

Before setting out to describe the model, it is worth elaborating that one problem with interpreting the output of any self-organizing system is that the output does not correspond to something easily interpretable. With a supervised system you instruct certain neurons to recognize certain inputs (e.g., neuron 1 should recognize images A to E). As a result you can assess network performance by seeing how often the designated neuron wins (e.g., how often neuron 1 responds most strongly to images A to E). In the case of a self-organizing system, the requirement is that the input space be divvied up in some useful manner, but the precise details are left to the system itself. So how are we to interpret the output of the system? Somehow or other we need to reverse-engineer the solution to comprehend it. In this section and next, various methods will be employed to achieve this and to use the network's representation of the input space to predict classification performance in behaviorally relevant contexts.

### The model

The model used in this initial set of simulations represents a very simple form of self-organizing competitive network model, a model which can be traced back to some of the earliest models of self-organizing neural classifier systems (von der Malsburg, 1973; Fukushima, 1975; Grossberg, 1976; Hertz et al., 1990). In its current form, it was recently used to describe how a feature-based system could explain the prototype effect (Wallis et al., 2008).

The network can be formally summarized as follows:

$$\begin{array}{l} \gamma_{ij}=\sum_{k}x_{k}w_{ijk}\\ \mu_{ij}=r{\left(\frac{N-\eta_{ij}}{N-1}\right)}^{\alpha }\\ y_{ij}=\frac{\gamma_{ij}-\kappa \gamma_{\text{av}}}{\gamma_{\text{max}}-\kappa \gamma_{\text{av}}}\\ \epsilon_{ijk}=\mu_{ij}y_{ij}x_{k}+(1-\mu_{ij})w_{ijk}\\ w_{ijk}=\frac{\epsilon_{ijk}}{\sqrt{\left(\epsilon_{ij}.\epsilon_{ij}\right)}} \end{array}$$

where *x*_*k*_ is the *k*th element of the input vector x, *w*_*ij*_ is the synaptic weight vector of the *ij*th neuron, and γ_*ij*_ is the neural activation of the *ij*th neuron. *N* is the number of classifiers (i.e., neurons) and η_*ij*_ is the rank of the neural activation, such that the most active neuron has rank 1 and the *n*th most active has rank *n*. μ_*ij*_ is a scaling factor based on a learning rate *r*, and the rank of the neural firing, which implements one aspect of global competition within the inhibitory pool of neurons. In the simulations that follow α was set to *N*, which had the effect that the ratio of learning in the second most active neuron was approximately one third that of the most active neuron. *y*_*ij*_ is the neural output of the *ij*th neuron, which is affected by the neural activation γ_*ij*_, γ_max_ which is the output of the most strongly firing neuron, and γ_av_ which is the average activation of the top ten most active neurons (excluding the neuron itself if it is in the top ten). Subtracting γ_av_ introduces a small amount of activity specific inhibition which further encourages neurons to select for inputs that are different to those selected for by other neurons. The constant κ was set at 0.3 in these simulations. Dividing by γ_max_ normalizes activity across the network on each stimulus presentation, which has the effect of ensuring that the amount of synaptic modification of the most strongly firing neuron is roughly constant for each image presented. This normalization step also implements a second form of global competition. The last two equations describe a form of standard Hebbian learning. The final equation normalizes the weight vector to unit length, the purpose of which is to constrain the size of the synaptic input weights. In effect enhancement of a specific input comes at a matched cost to other synaptic input lines. Although the precise mechanism behind such weight distribution are unknown there are theories suggesting that this might be part of the functional role of sleep (Crick and Mitchison, 1983; Hinton and Sejnowski, 1986; Bushey et al., 2011).

In this simple model the neurons are afforded just three inputs (i.e., *k* = 1, 2, or 3) meaning the weight vectors lie on the surface of a sphere of unit radius. As all weights are also constrained to be positive, the weight vectors all lie in the positive octant of a unit sphere. Note that a double subscript “*ij*” is applied to the output neurons to afford them grid co-ordinates corresponding to their placement within the cortical surface. Although not important in this initial simulation, the importance of spatial neighborhoods described by these coordinates will be become apparent in a later section investigating the effects of lateral excitation.

More powerful models of associative behavior than Hebbian are clearly possible (such as covariance learning). But in a sense, if Hebbian learning suffices, we know the brain has more power at its disposal since Hebbian association can be regarded as a subset of what its neurons are really capable of. Overall, although the network implementation may seem obscure, it is important to bear in mind that this is a biologically relevant implementation of a system designed to divvy up a two-dimensional input space in a manner exactly like that described in Figure 1. That figure does, in fact, represent the output of this same network.

### Holistic processing

As a first step it is important to reiterate how I am conceptualizing holistic processing. I, and many others, have argued for a representation of objects and faces on the basis of abstract features (Poggio and Edelman, 1990; Wallis and Rolls, 1997; Wallis and Bülthoff, 1999; Ullman, 2006; Torralba et al., 2007; Wallis et al., 2008). I regard holistic processing as evidence for multi-feature (i.e., multi-dimensional) selectivity. Rather than just being interested in the presence of a nose, or the distance between the eyes, or hair color, or any other (more abstract) single feature of a face, I am arguing that under certain circumstances neurons will seek out multiple dimensions of the input, making the neuron highly selective for a few stimuli and highly sensitive to changes in any one of a number of features. This approach differs from arguments based on strictly holistic features such as those generated by principal component analysis (O'Toole et al., 1991; Cottrell and Hsiao, 2011), since neurons can be tuned to a single nameable feature and anything in between.

If one accepts my characterization of holistic processing, understanding how holistic processing comes about demands an understanding of how neural classifiers in a competitive system switch from low-dimensional to higher-dimensional selectivity. The proposal I have been building to, is that it is governed by the number of neurons competing to represent a particular region of input space. To demonstrate this effect, simulations of the model system described above were run.

**Figure 2:**
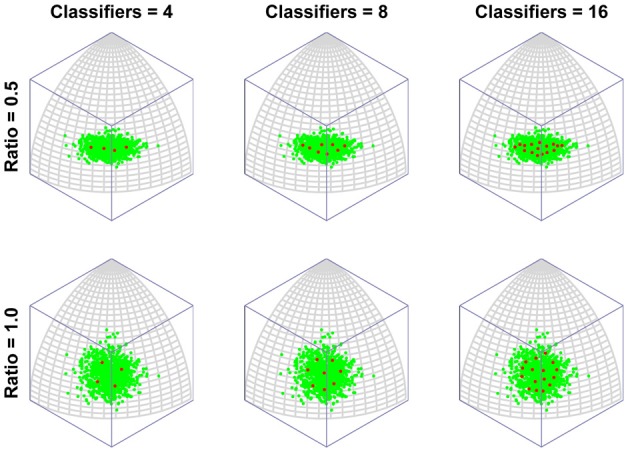
**The emergence of multidimensional (holistic) selectivity in neural classifiers.** The six plots represent outcomes of the simulation. From left to right the number of neural classifiers increases. From top to bottom relative levels of variance in two orthogonal dimensions of face space are tested. The variable “Ratio” controls the ratio of the standard deviation of the less variable dimension (Dimension 1, running along lines of longitude) to that of the fixed dimension (Dimension 2, running along lines of latitude). For large discrepancies in variance and small numbers of classifiers, the classifiers align with Dimension 2, completely ignoring variation in the minor dimension. Increasing the number of classifiers or the variance in Dimension 1, sees classifiers becoming sensitive to both dimensions.

Figure 2 depicts the outcomes of six simulation runs. In each case 500 exemplar inputs were chosen which varied along two orthogonal dimensions. The ratio of variance of the two input dimensions was varied as well as the number of classifiers. The figure shows the steady-state outcome of the simulation with the following parameters: (*r* = 0.001, *N* = [4, 8, or 16], α = 4). The main message of the simulation is that for small numbers of neural classifiers the tendency is for neurons to become selective along a dimension of high variance. Of course this is a perfectly valid means of dividing up the input space evenly, but it has the interesting consequence that the neurons are indifferent to the input dimension of lower variance. Once variance is matched (Ratio = 1) the neurons spread out evenly across the two dimensions, but it seems that when only few neurons are active in an area of input space, even a modest discrepancy in the amount of variance between dimensions of the input space can result in neurons exhibiting low-dimensional (in this case single-feature) sensitivity.

In contrast, even if the dimensions are mismatched, by increasing the number of neural classifiers, mutual inhibition forces the classifiers off the axis of highest variance, producing a broader spread of classifiers tuned to multiple dimensions of the input.

**Figure 3:**
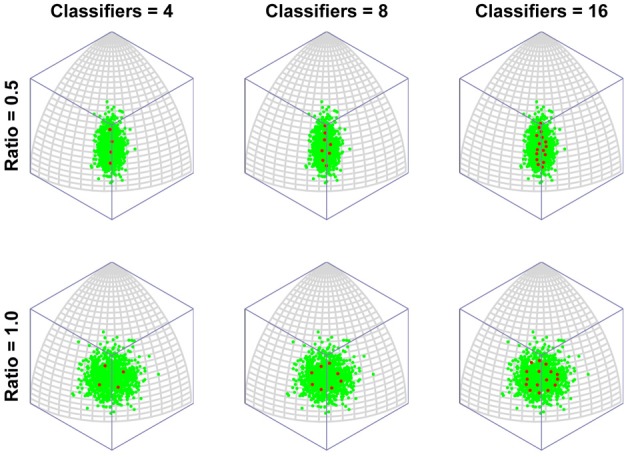
**The emergence of multidimensional (holistic) selectivity in neural classifiers.** The figure is exactly comparable to Figure 2 except the axis of maximum variation is switched from longitude to latitude to demonstrate that the effect is not somehow being distorted or exaggerated by plotting on the surface or a spherical under orthogonal projection.

To put this in more biologically relevant terms: in the absence of competition a neuron will become tuned to a feature that it sees regularly. If faces containing that feature are seen often enough, other neurons will also seek to respond to that feature. In the end, the feature will be shared by several neurons and, in the process, the feature will lose its potency for driving learning, as its mere presence will no longer guarantee that a neuron always fires (due to competition with similarly tuned neurons). Neurons will then tend to concentrate more of their resources (synaptic weight) on other, less common features. This is the route to more integrative, holistic processing. As the number of classifiers increases, so the tendency increases to focus ever more resources on ever more minor feature dimensions. In other words, the process of recruiting ever more neurons to a region of face space leads to that region being represented by neurons tuned to an ever more holistic array of features of the face. Figure 3 presents the same results but with the axis of maximum variation switched, to demonstrate that the organization of classifiers is not in some way distorted by the method of projection used (i.e., an orthographic projection of a spherical surface).

Note that the model yields holistically tuned neurons when highly over-trained on a set of stimuli with small variance. This can be seen as an explanation for why we have holistic representations of upright faces (seen often) and not inverted ones (rarely encountered). But because these effects are a result of learning, the model predicts that orientation-sensitivity effects can change with appropriate experience. There are at least two relatively recent papers that support this idea:

- The face inversion effect can be overcome with learning (Laguesse et al., 2012).
- With sufficient training to a particular orientation, non-face objects develop inversion effects (Husk et al., 2007).

In a similar vein, one study looking at face adaptation after-effects was able to show that it is possible to obtain simultaneous and opposite adaptation to upright and inverted faces in FFA, suggesting that the neurons representing the faces are separate (Rhodes et al., 2004). The authors took this as evidence for separate populations supporting the analysis of faces in the two views. They went on to suggest that one population performs featural analysis of inverted faces, whereas the other performs holistic processing of upright images. This is consistent with the model described here if one regards inverted faces as somewhat akin to other-race faces, in the sense that they are rarely encountered and hence sparsely and separately represented from upright own-race faces. An alternative explanation, perhaps more consistent with the data of Yovel and Kanwisher (2005), is that inverted faces weakly activate incorrect holistic representations in FFA which can be adapted independently of the correct (and hence separate) holistic representations activated when seeing the same face upright. An explanation along these lines would also be consistent with the model being proposed here.

A final point worth making about the model described here is that there is no “simulated annealing” or other form of gradual learning rate reduction used, as was common to many self-organizing models in the past. As such the model is capable of comprehensive restructuring as the distribution of inputs evolves or suddenly changes. There is actually some evidence for this in humans in the form of the changing size of the FFA (Golarai et al., 2007; Scherf et al., 2007). Studies of monkey temporal lobe cortex have likewise described how focussed training on a novel stimulus set can generate large numbers of neurons selective for the new stimulus class (e.g., Miyashita et al., 1993; Logothetis and Sheinberg, 1996; Baker et al., 2002). As mentioned in the introduction, some of the best behavioral evidence for continued restructuring and learning of face processing comes from the study of recognition in same and other-race faces, and that is what I turn to next.

### The other-race effect

For those who remain unmoved, this section forges a more concrete link between the classifier network's behavior and measurable human behavior. As mentioned above, interpreting the actions of a self-organizing system can be non-trivial, but this section offers a means of directly testing how discrimination performance varies as a function of the distribution of classifiers which the system produces.

In the introduction I argued that if an observer spends a large amount of time looking at a particular region of object input space, more and more neurons become recruited to that region of space and the observer will tend to develop ever more holistic representations of the inputs as a result. Presumably, therefore, the model should reflect the density of inputs in each region of input space, as well as the relative frequency of their occurrence. One way to test this with the model is to introduce two populations of inputs with different centers of mass and different numbers of exemplars, that is, two populations of faces which differ along one or more feature dimensions and for which we have differing levels of exposure. Far from an obscure theoretical thought experiment, the situation I am describing is none other than the basic ingredients of the other-race effect.

There are good reasons for thinking that the model described here can explain the other-race effect because a related approach has been successfully applied already. Over several years, O'Toole, Abdi and collaborators have advocated an approach based on features derived from principal component analysis (O'Toole et al., 1991; Furl et al., 2002; Bartlett et al., 2003; Caldara and Abdi, 2006). In their model the classifier features are allowed to emerge from the stimulus set in much the same way that the abstract features promoted in this paper are. Their work then demonstrates how such a feature-based representation, “warped” or “molded” by the input, naturally generates an other-race effect (O'Toole et al., 1991; Furl et al., 2002). The precise mechanisms by which such a model might be implemented in cortex are left largely unexplored (Cottrell and Hsiao, 2011), but in many ways that is not the goal of the work. The authors generally use the PCA-features as a front-end to a classification network trained using supervised methods to prove the in-principle relation between the other-race effect and a feature-based representation. As with Lewis et al.'s work, such an approach raises the question of the impact of this layer of supervised training in which the network is externally forced to focus learning on same-race faces. The point of the simulations described here is to take the next step and offer a more biologically relevant, self-organizing system which also speaks to recently reported links between holistic processing and the other-race effect (Michel et al., 2006) which I discuss below.

**Figure 4:**
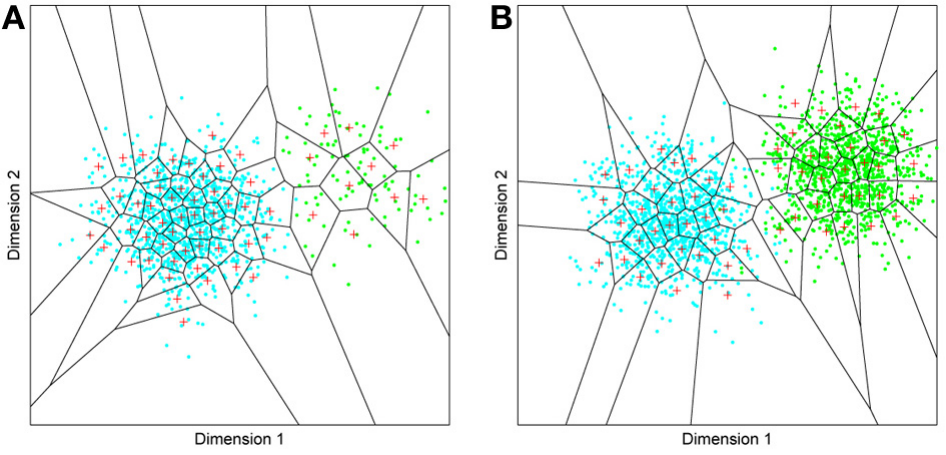
**Effects of differing levels of exposure to two subsets of faces.** Each “Dimension” could correspond to nameable features such as nose width, hair color, head shape etc., but they might just as well be complex combinations thereof, and hence be hard to characterize in words. The “+” points represent the distribution of neural classifiers which emerge from competitive learning of the faces. In other words the “+” markers indicate the neural weight vector of the 50 neurons which serve as the set of abstract-feature classifiers. Light blue dots represent the locations of a frequently viewed category of inputs (e.g., own race faces) in the space, and green dots those of inputs viewed less frequently (e.g., other race faces). **(A)** With 1000 faces from the observer's own race and 100 from another race, the number of classifiers covering the space occupied by own-race faces is much higher and much more compact (holistic and highly discriminating) than in the other-race region of the input space. **(B)** Balancing the numbers of exemplars in each race (1000 in each) produces equivalent distributions of classifiers for the two races.

But before reviewing that link, we can start by asking whether different levels of dimensionality and sensitivity emerge in the system when there are changes to the relative frequency with which sub-regions of face space of faces are experienced. The results of such a simulation are displayed in Figure 4A, which is the outcome of a simulation of the same network with two distributions of faces of equal variance but different rates of occurrence (50 neurons, 100 faces from a rarely encountered group of faces, and 1000 from a regularly encountered group of faces). If the number of faces encountered in each race is matched, the sensitivity bias disappears, see Figure 4B. Note that in this case I have elected to transform the three-dimensional weights vectors and inputs into two flat dimensions. It's easier to read and interpret that way and loses no information since the weights are restricted to two degrees of freedom moving around the surface of a sphere. The new dimensions correspond to the azimuth and elevation of each vector. This is like the projection of the world onto a flat surface, much as the world map can be flattened onto a page^2^.

It is apparent from Figure 4A that the model system does indeed produce the expected type of behavior. In the region of many exemplars (same race) the network produces holistical classifiers with selectivity tightly tuned along both dimensions. In contrast, the region of more sparse inputs (other race) produces relatively few, more broadly tuned neurons.

**Figure 5:**
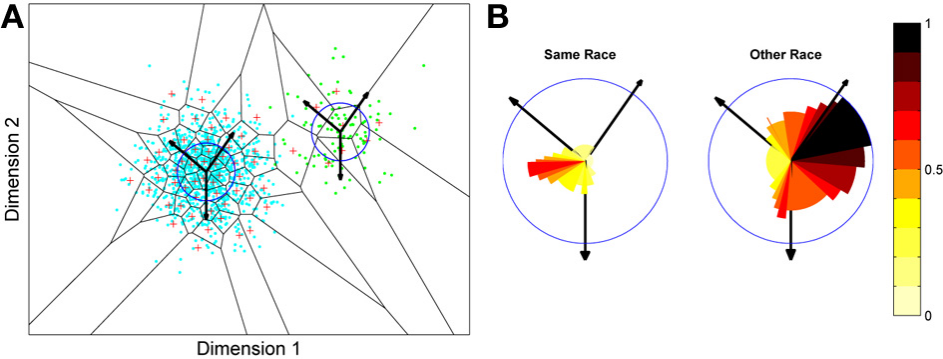
**Measuring face discrimination ability of the network. (A)** The original face space with distributions corresponding to same and other-race faces (in light blue and green dots, respectively). The circles represent 1 s.d. from the center of each of the two distributions of faces. The arrows represent three example trajectories from the mean along which discrimination performance was measured. **(B)** Distractor rejection ability of the network measured as the number of steps taken (in 0.1 s.d. steps) before the network was able to discriminate the new face from the mean face image. The small amplitude of the cut-off for same race faces across all trajectory orientations is indicative of better discrimination performance by the network in the region corresponding to the same race face.

What we can now do is check whether the network's organization of the input has resulted in the types of behavior that typify the other-race effect in humans, namely relatively poor discrimination performance for other-race faces. To test this, a series of new “distractor” faces can be generated which differ from previously seen images by an objectively measured amount. The prediction is that the model will be less able to distinguish the distractors from a known face if it falls within the sparsely represented area of other-race faces, compared with performance on distractor faces falling within the same-race region of space. To test this the center point of the same race faces was measured and the response of the network recorded. Then a new image was presented which differed by 0.1 standard deviations from this mean. If the same neuron responded most strongly to this face as responded to the mean face, then this was taken as a failure of the network to discriminate the two faces. A new distractor was then presented to the network differing by 0.2 s.d. from the mean face, and again the response was checked to see if now a different neuron was responding. This process was repeated until the winning neuron changed, or 1 s.d. was reached. This process was also repeated for all combinations of the two feature dimensions, forming a series of trajectories through the space radiating from the mean. The trajectories covered all 360° of a circle in 1° increments. The same process was then carried out on the other-race faces, based on trajectories radiating from the mean of the other-race faces. The results of the analysis appear in Figures 5A,B^3^.

So, the network has replicated the fundamental result of the other-race effect: discrimination is worse for other-race than own-race faces. From these simulations it also becomes apparent that the model predicts an intricate interplay between the other-race effect and degree of holistic processing. For example, because same-race faces are regarded as objects of expertise they should be represented more holistically. The model makes a number of predictions, many of which have indeed been confirmed in the behavioral literature of the past 6 or 7 years:

- Observers process own-race faces more holistically than other-race faces (Michel et al., 2006; Rossion and Michel, 2011; DeGutis et al., 2013)^4^.
- The extent to which faces are processed holistically predicts face discrimination performance (Richler et al., 2011).
- Observers show a discrimination advantage for sub-populations other than those of their own race, such as their own age-group (Hills and Lewis, 2011; Hills, 2012). The model predicts this if one assumes people tend to mix with people of their own age and hence are exposed to them more often than faces of other age groups.
- Increased exposure to faces of any particular age (e.g., teachers with children) produces more holistic processing of faces in that age group (de Heering and Rossion, 2008).
- The other-race effect can be overcome with experience (McGugin et al., 2011).
- Familiar other-race faces can be subject to holistic processing (McKone et al., 2007).
- The other-race effect can be reversed by a complete change of racial environment, at least in children (Sangrigoli et al., 2005).

Incidentally, Valentine (1991) suggests that exemplar-based models can also explain the “other race *advantage*”—i.e., that observers are quicker and more accurate at detecting the race of a face if it is taken from another race than from their own (see also Valentine and Endo, 1992; Zhao and Bentin, 2008). On the basis of the model as it stands, there are two potential sources of explanation:

On the one hand, the effect may be due to the action of a decision layer subsequent to the level of representation described here. This layer would support generic classification of the faces along numerous dimensions (age, gender, race etc.). The issue of which categories are most easily activated would be governed by which categories a person most often accesses. To put this another way, one can consider levels of categorization. The category entry level for other-race faces is quite likely at the level of race, whereas for own race faces it is at the level of the individual. It is well known that accessing superordinate or subordinate representations takes time (Rosch et al., 1976), hence it may be the case that it is the matching of task to natural (entry-level) categorization that promotes the task-specific, other-race recognition advantage. In favor of this interpretation, we know the details of an observer's task affect the extent to which holistic processing develops. Basic-level categorization of objects produces skill at dissociating at the level of groups, whereas training on individuation enhances the holistic nature of the representation and, with it, reduces performance on group discrimination (Wong et al., 2009a), and several parallels have been found in face processing too (McGugin et al., 2011). Note that this result suggests that the higher categorization process may feed back to the recognition system, encouraging the formation of holistic vs. less holistic representations according to task demands. If so, that is something beyond the scope of the current model.

An alternative explanation, which does not invoke the actions of a later decision process, emerges from the fact that other-race faces are represented by neurons with generic, less-holistic tuning. Consider the fact that if a generic marker of race (such as skin color or eye shape) exists; for other-race faces this single feature is likely to be adopted by a neuron sensitive to that region of the input space. What is more, the neuron will tend to put all of its neural resources into responding to that single feature as it is the one feature which all faces in that area contain. In the own-race face region of input space, that generic feature will be available too, but due to the crush of neurons tuned to that generic feature, it will not be sufficient, in and of itself, to produce a reliably strong response in a single neuron. Hence in this case, the neuron will distribute its neural resources over other, more specific features, rather than the more generic, race-related one. This type of explanation finds echoes in the ideas of Cottrell et al. (Haque and Cottrell, 2005).

As a final aside, one might ask why I am arguing that high-dimensional representations underlie the composite face effect, when any number of single abstract but holistic feature (e.g., head shape) would suffice. What my model offers is an explanation for why holistic processing emerges in faces of expertise (own race faces) and not in other races. Own-race face classifiers are multi-dimensional (and so probably the vast majority include at least one feature which spans the upper and lower halves of composite faces), whereas other-race neurons will tend to be selective to only a few features, increasing the likelihood that these happen to be features which do not span the two face halves. Evidence for this line of reasoning will be provided in the next section.

## A model of face recognition

### Introduction

The previous section has described a simple, self-organizing model of object representation which was seen to be able to explain the emergence of holistic and other-race effects in a category of expertise. The main problem with models of this type is that they are largely conceptual and their relevance to real-world recognition tasks can appear obscure. In this section the concepts developed above will be applied to a more biologically relevant model of face processing, permitting testing of other well-established behavioral effects.

In common with numerous models of recognition in inferior temporal lobe cortex, the new model is an appearance-based model, deriving input from abstract visual features tuned to reflect the statistics of the visual environment (Wallis and Bülthoff, 1999; Ullman, 2006; Wallis et al., 2009). Further, and in common with numerous models of object recognition, the model is organized into multiple layers of competitive networks (Fukushima, 1988; Wallis and Rolls, 1997; Riesenhuber and Poggio, 2000). This section considers patterns of selectivity which emerge in such a system after exposure to an array of facial images.

### The model

The model itself is based loosely on biological principles, although no attempt is made to explain object constancy i.e., view invariance. For solutions to that problem see the review on temporal association learning referenced earlier (e.g., Wallis et al., 2009). Instead, the input faces are all processed at the same location and scale, and are then transformed into localized, edge-based representations by passing the image through a Laplacian of Gaussian filter followed by a Gaussian filter, to smooth the output (s.d. = 5 pixels). The output is then amplitude normalized to the range 0–1.

Edge detecting the images represents an attempt to mimic the filtering properties of simple cells known to reside in primary visual cortex (Hubel and Wiesel, 1977). This places the emphasis on differences in high-frequency content of the faces. As an aside it is important to note that this is only part of the story. Real faces vary across many spatial scales and recent results from single cell recording have highlighted the importance of contrast across broad patches of the face (Ohayon et al., 2012). A more complete description would include filters of differing spatial scales like those found in primary visual cortex and already incorporated into hierarchical models of vision (e.g., Mel, 1996; Wallis and Rolls, 1997; Itti and Koch, 2001). One important advantage of processing images across spatial scales is that it allows later neurons to discover frequency bands across which their primary stimuli differ, which in the case of faces appears to be biassed toward lower frequencies (Keil, 2008). Since these differences are intrinsic to faces, a self-organizing system would naturally tune face selective cells to lower frequency bandpass filters (Keil et al., 2008). This low frequency bias is regarded by some as the driving force behind the holistic processing of faces, a proposal which they have backed up through behavioral experimentation (Goffaux and Rossion, 2006; Awasthi et al., 2011). I would nonetheless argue that although the bias may be a contributing factor, and one which a self-organizing system could replicate, it does not offer a simple explanation for why other-race faces are processed non-holistically (Michel et al., 2006), since they should possess a comparable spectral bias to own-race faces.

With this caveat aside, the rest of the model operates like many other hierarchical models of object recognition. The filtered input is sampled by groups of neurons operating in mutually-inhibitory (competitive) pools, which act to divide up the limited extent of the input to which they have access. In the subsequent decision layer, a fully connected system of neurons compete with one another to represent the input space of faces. Central to the network's design are three core elements which it shares with all self-organizing, competitive systems: (1) A mechanism for neural integration of its inputs. (2) A rule for synaptic adaptation which in this case is based on simple Hebbian principles. (3) A form of mutual inhibition implementing competition between neural classifiers within a mutually connected pool (Hertz et al., 1990; Wallis and Rolls, 1997).

The network's first layer is subdivided into 16 inhibitory pools arranged in a 4 × 4 grid, with each pool containing *N*_1_ = 9 neurons. Activation of the *i*th neuron γ_*i*_ within an inhibitory pool, is the product of its corresponding weight vector *w*_*ijk*_ and the current input vector *x*^*ab*^_*k*_. The neuron's response *y*_*ij*_ is then a result of its activation and the level of inhibition from other neurons within the inhibitory pool. In layer 1 each neuron within a pool samples from a 16 × 16 pixel array extracted from the corresponding 16 × 16 pixel section of the input image. The second layer contains a single, wholly laterally connected network of *N*_2_ neurons which sample the entire set of layer 1 neurons across all 16 inhibitory pools.

The network can be characterized by the following set of equations:

γij=∑kxkabwijkμij=r(N−ηijN−1)αyij=γij−κγavγmax−κγavϵijk=μijyijxkab+(1−μij)wijkwijk=ϵijk(ϵij×ϵij)

**Figure 6:**
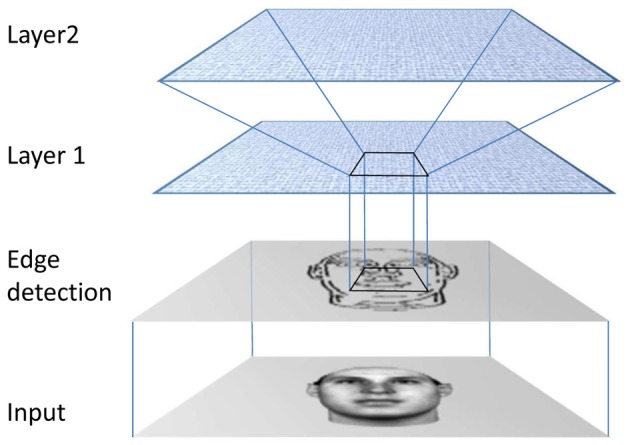
**The network processes the input image in three stages.** The first stage performs edge detection. Output from this stage is then read by the first neural stage. Processing in this stage is localized such that a pool of interconnected neurons samples a corresponding patch of the edge-detected image. The final stage is Layer 2, which contains a single pool of interconnected neurons which all receive input from the entire array of Layer 1 neurons.

Overall the equations are identical to those used in the network model described earlier. The superscript *ab* is attached to the input *x* to indicate that only a subregion of the input is seen by neurons within a specific pool. In this case there are 4 × 4 pools meaning *a* and *b* vary in the range 1–4, corresponding to the 16 subregions of the input image. In layer 2 the input vector is simply the entire output of layer 1 across all 16 inhibitory pools and all nine neurons within each pool. In self-organizing systems graded inhibition, of the type being used here, has been shown to encourage a smooth representation of the input space (Bennett, 1990). In this case discontinuities may still arise due to discontinuities in the input space itself (such as occur between object categories). The general network architecture is depicted in Figure 6.

### Simulations

**Figure 7:**
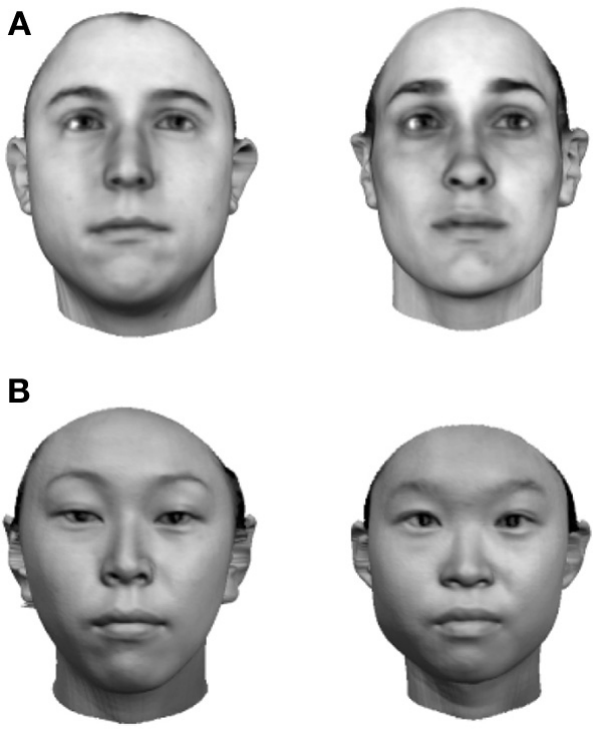
**Input face images. (A)** German female faces. **(B)** Japanese female faces.

The network was trained using faces taken from the Max Planck database of 3D scanned heads, see Figure 7, and rendered at a resolution of 256 × 256 pixels. Forty-three German and seven Japanese female faces were presented. Each image was presented once in pseudo-random order, and the process then repeated a total of 100 times. During this initial exposure, only layer 1 neurons altered their synaptic weights. The learning rate parameter *r* was set to 0.001 and the ranking inhibition parameter α was set to the pool size, i.e., 9. Once learning was complete in layer 1, the same procedure was followed but now allowing layer 2 neurons to learn, in this case with the same value of *r* but α set to 1. Offsetting learning in the two layers was done mainly for convenience. Allowing layer 1 to converge first ensured that learning in layer 2 was conducted on a stable platform, allowing layer 2 learning to converge more quickly. Learning in layer 2 was run for 300 iterations of the complete stimulus set. The network was tested with 5, 10, 25, 50, and 100 outputs, which all produced qualitatively similar results. The figures here all represent data from the system for 10 classifiers, i.e., 10 output neurons.

**Figure 8:**
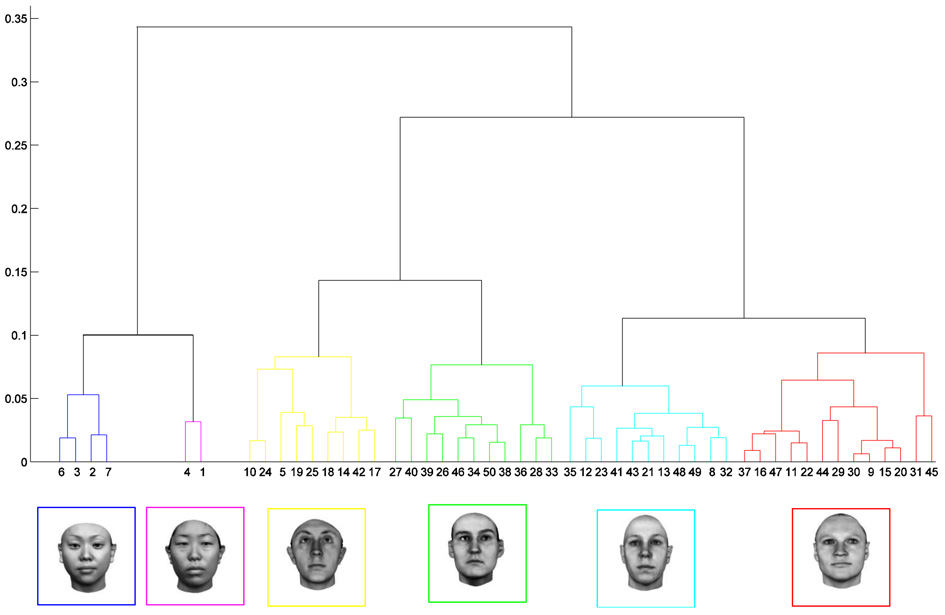
**Cluster analysis of the neural output from the network represented in graphical format.** An example face from each of the six major clusters appears along the horizontal axis. There is a clear tendency for the system to produce consistent responses to subsets of the input stimuli. Of particular note is the differentiation of German and Japanese faces. The difference represents the primary bifurcation of the dendrogram, suggesting little overlap in the groups of neurons responding to the two racial groups.

Given the aforementioned difficulty of measuring performance of a self-organizing system, how can we approach it in this case? One possible starting point is provided by the fact that, in this case, the system has been trained with a large number of Caucasian faces, and relatively few S.E. Asian faces. We can begin by asking how the neural responses of the output neurons differ across stimuli and, specifically, race. To do this a standard cluster analysis approach was applied to the output firing rates of the layer 2 neurons for all 50 learnt faces, based on the Matlab “dendrogram” function (using Ward linkage, Euclidean distances, and six clusters). The results of the analysis appear in Figure 8. Note that six of the seven Japanese faces cluster under two closely related nodes. This emergent clustering behavior cannot simply be ascribed to skin-tone differences or indeed any other trivial luminance effects because the input images were high-pass filtered and amplitude normalized before being provided as input to layer 1 (see the earlier description of the edge detection phase).

**Figure 9:**
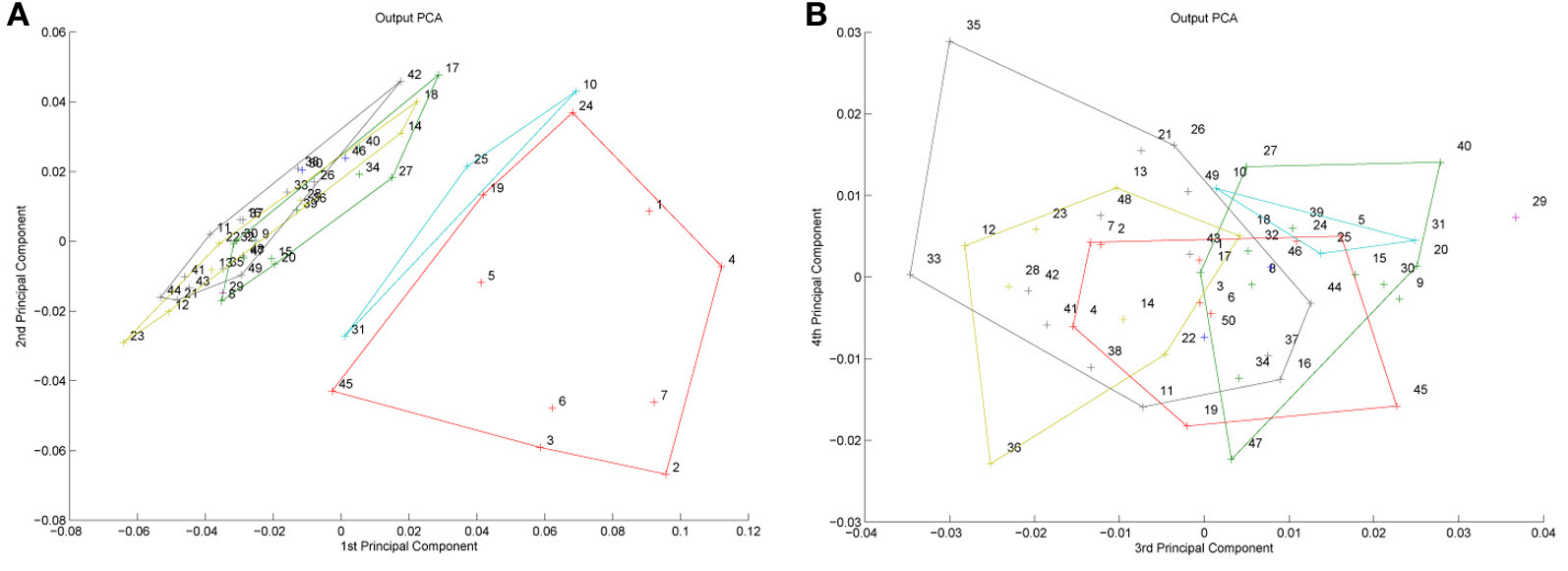
**(A)** Output of the network projected onto its principal components. Outputs are labeled by a code corresponding to the specific input image which generated the output. Image numbers 1–7 correspond to the other-race faces. The colored “+” and corresponding subregions of output space represent the region of the space for which a specific neuron wins. Note that for the first two principal components, the other race faces are strongly separate from the same-race ones, and a single neuron has learnt to respond to any of these other-race faces. The subregion corresponding to same-race faces is, by contrast, much more densely packed and neural regions of selection heavily overlap, suggesting that other, higher dimensions play an important role in determining which neuron dominates for each input. Note that face 5 was the only other-race face to be included in a branch of the dendrogram selective primarily for own race faces. The reason becomes apparat from this figure in that face 5 lies on the boundary between own and other-race faces. **(B)** The same output data projected onto components three and four.

The issue this type of analysis is trying to resolve is how best to visualize the high-dimensional space described by the neural output. An alternative method of visualizing high-dimensional data is to conduct a principal component analysis. The responses of the system to each of the 50 inputs can then be pictured, projected onto the major dimensions of variation within the output. An analysis of this type is shown in Figure 9. The message from this analysis is that the network has created one neuron which is cornering a large region of space containing relatively few, related images (which correspond mainly to the other-race faces). At the same time the network has placed far more neurons in the more densely packed region of space containing the 43 same-race faces. As a small caveat, I should add that it is wise to be a little wary about over-interpreting the figure. The output space is generated by the network and does not correspond in any simple manner to the original input space of images.

Another means of assessing performance of the network is to check its ability to create a unique pattern of firing for each stimulus, i.e., its powers of discrimination. In this case a unique code can simply be defined as a pattern of neural activity in which the ranked order of neural outputs is unique for that particular input. With 25 output neurons the network was able to produce a unique code for all 25 inputs. Even with as few as 10 neurons it created unique output for 35 of the 50 stimuli. Interestingly, due to the low coverage of the region of space corresponding to the other-race faces, the network correctly discriminated just 28% of the other-race faces in this case. In contrast, the rate for own-race faces was 75%.

**Figure 10:**
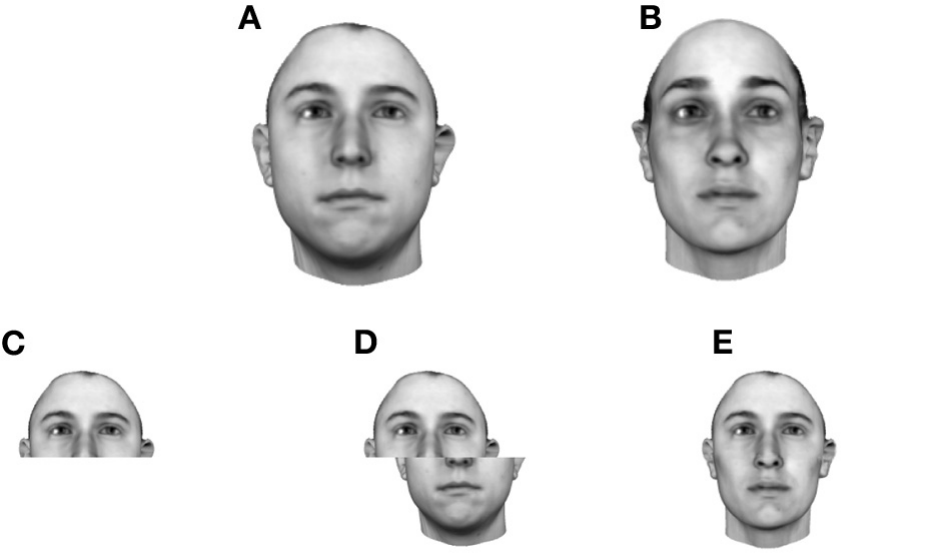
**Face manipulations designed to test the holistic processing of learnt faces. (A)** Face A. **(B)** Face B. **(C)** Face A lower half deleted. **(D)** Face A, lower half horizontally offset. **(E)** Face A with the lower half replaced with the lower half of Face B.

So, it appears that the system has learnt to represent the faces, and in a manner that allows for image discrimination as well as a sense of intrinsic image similarity. But how does this relate to holistic processing for example? A more direct test of the sensitivity of the classifiers to holistic cues is to look at changes in their response to disruptions in the holistic form of the input faces. Examples of possible image manipulations appear in Figure 10. Because I regard holistic processing as a function of expertise, I am predicting that neurons responding to the Caucasian faces will produce more holistic representations of their preferred stimuli than the Japanese faces. In other words, the expectation is that faces falling into the other-race category will exhibit less sensitivity to changes in appearance of the stimuli than those of the same race, for which many more exemplars have been seen. To test this hypothesis the trained network was exposed to two sets of manipulated faces in which either the lower half of the image had been deleted, or replaced with that of another face.

The question then arises, how can we assess the impact of these image manipulations on the network? One possibility is to look at the effect it has on the most active neurons (which presumably encode the identity of that face). If the range of neurons which are active remains the same pre and post image manipulation, the system is tolerant to such manipulations and the representation could be thought of as non-holistic. Conversely, if the pattern of most active neurons changes a great deal, it suggests that the neurons coding for the original (unmanipulated) face image are sensitive to that manipulation. This, in turn, suggests that they are sensitive to information from various parts of the face and hence are encoding the face more holistically.

**Figure 11:**
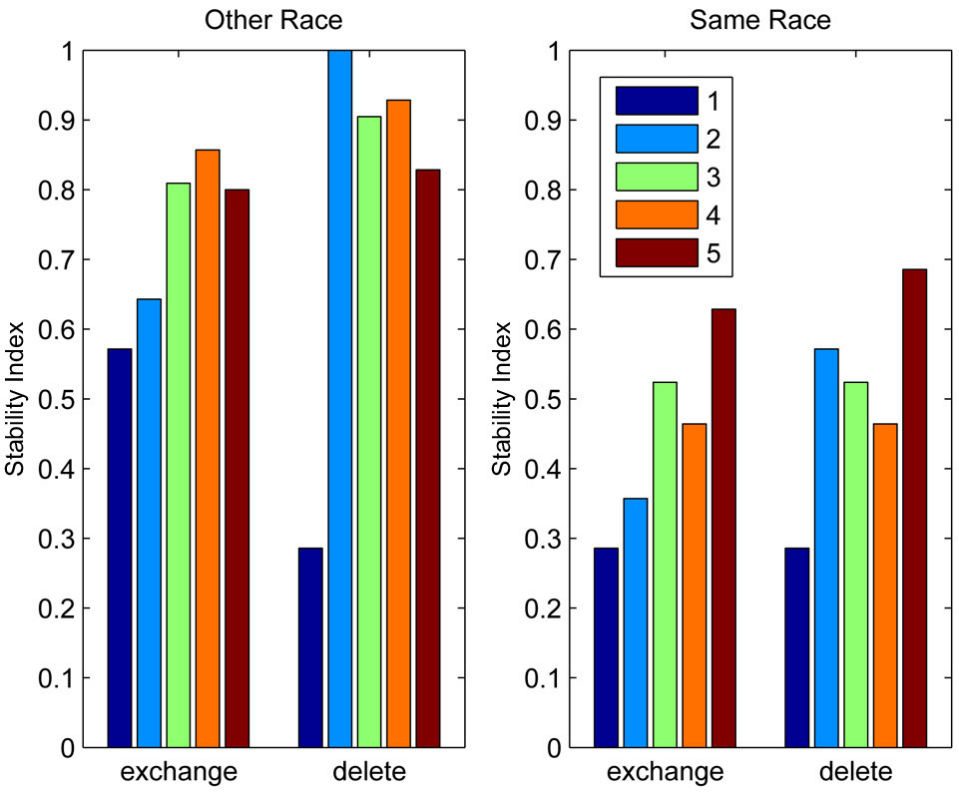
**The stability of the network response to face stimuli undergoing two types of image manipulation.** The “exchange” condition corresponds to the replacement of the lower half of a face with that of another person. The “delete” condition corresponds to the blanking of the lower half of the image. The vertical axis provides a measure of the overlap in neural output activity between the control condition of the original stimulus being presented and the particular manipulation, such that 1 indicates that exactly the same “n” neurons were most active after manipulation of the image as were most active before. Analysis of just the most active neuron is labeled “1”, the two most active neurons labeled “2”, and so on up to the five. As one might expect, deletion is seen to produce smaller changes in the output code from the control condition than exchanging the lower half of the face with another. Significantly, the impact of both manipulations is much more marked for the same race faces than for the other race faces (output similarity drops well below 1.0).

To test this, the output of layer 2 was analyzed to see how much the response of the system was affected by two types of stimulus manipulation. Analysis involved taking the top *n* most active neurons and asking whether the same *n* neurons were active to the manipulated version of the face. Results appear in Figure 11 divided between same and other races with data averaged over seven faces selected at random from each race.

The model clearly produces classifiers of the more familiar own-race faces that are, on average, more likely to be sensitive to changes to the whole face than classifiers focussed on representing the other-race faces.

## A model of cortical organization

### Introduction

As a final stage to the modeling work, this section considers the issue of cortical patterns of neural selectivity. Over the past 5 years or so, more and more evidence has emerged supporting the view that the ventral visual stream contains areas dedicated to the processing of facial stimuli. As mentioned earlier, Doris Tsao et al. have taken the lead in this endeavor, describing the presence of an entire hierarchy of face selective “patches” which demonstrate steadily increasing levels of tolerance to changes in viewpoint of their preferred stimuli as a function of their location through the visual hierarchy (Freiwald et al., 2009; Freiwald and Tsao, 2010) (see also Rolls, 1992; Barlow, 1995). Although mainly focussed on the study of monkeys, parallels with humans have also been investigated and verified (Tsao et al., 2008a). This work broadly supports earlier reports of clustering of neural selectivity across faces and objects throughout the temporal lobe (Perrett et al., 1984; Tanaka et al., 1991; Fujita et al., 1992; Wang et al., 1996; Zangenehpour and Chaudhuri, 2005).

### Lateral association

Up to this point, the models being proposed offer no explanation for how such patches occur. However, this is easily remedied through the introduction of short-range lateral excitation. It has been known for many years that short-range lateral excitation can produce large-scale smooth variations in selectivity similar to that described in many regions of visual cortex. Initially, lateral excitation was used to produce self-organizing systems that mimicked mappings found in early visual areas (e.g., von der Malsburg, 1973; Willshaw and von der Malsburg, 1976; Kohonen, 1982; Olson and Grossberg, 1998), but they have also been successfully applied to explaining the orderly arrangement of selectivity in higher areas (Michler et al., 2009). In general, these systems generate smooth maps, but only because they are driven by a smooth input space (disparity, spatial location etc.). Discontinuities in the maps can occur if the input is discontinuous (left vs. right eye) or if an uneven distribution of exemplars exists across the input space, as is certainly the case for objects.

In the case of the models described earlier, local interactions can be implemented by allowing neural activity to be influenced by the activity of immediate neural neighbors. The expression for neural firing becomes:

$$y_{ij}=\;\left(\frac{\gamma_{ij}-\kappa \gamma_{\text{av}}}{\gamma_{\text{max}}-\kappa \gamma_{\text{av}}}+h\sum_{ab}\frac{\gamma_{ab}-\kappa \gamma_{\text{av}}}{\;\gamma_{\text{max}}-\kappa \gamma_{\text{av}}}\right)$$

where *h* controls the relative contribution of the local horizontal excitatory connections. The variables *a* and *b* iterate across the immediate neighbors of the *ij*th neuron. In the simulations described here, lateral excitation was received from the eight nearest neighbors in the grid as a simple average. In other words *a* varied in the range *i* − 1 ≤ *a* ≤ *i* + 1 and *b* in the range *j* − 1 ≤ *b* ≤ *j* + 1. To demonstrate the impact of including local excitation, a new series of simulations were run in which the neurons were accorded a physical location across the cortical surface in a 2D grid.

**Figure 12:**
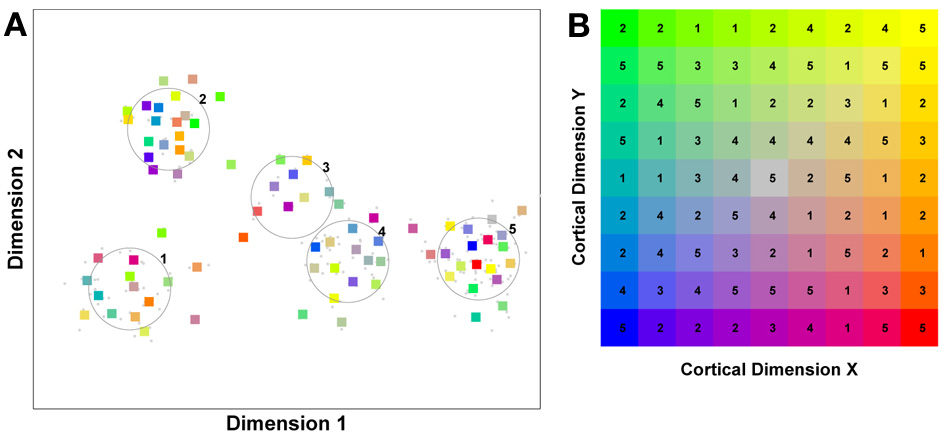
**Selectivity preferences for 81 neurons exposed to five object categories containing 50, 40, 20, 50, and 100, exemplars, respectively.** No lateral interaction between classifiers. **(A)** Each colored square represents a classifier whose color encodes its relative position across the 2D surface of cortex. The five circles represent the extent of the five object categories. **(B)** The color-based encoding of classifier location in cortex. Blue and yellow represent points on the cortical surface that are far apart, as do red and green. Physically proximity is conveyed by the change in color (hue). The number appearing on top of each neural classifier represents the object category for which that particular neuron is selective. Notice in this case that there is no evidence of any structure in the neural selectivity, i.e., there is no link between category and the physical location in cortex of the neuron.

**Figure 13:**
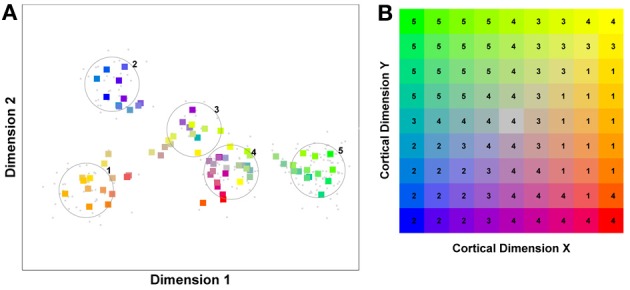
**Selectivity preferences for 81 neurons exposed to five object categories.** The classifiers were subject to moderate local lateral excitation (*h* = 0.3), but otherwise the simulation parameters were identical to those shown in Figure 12. Notice in this case that there is evidence for emerging structure in neural selectivity, i.e., object category and physical location are now loosely related. **(A)** Neural selectivity within object space. **(B)** Neural selectivity across the cortical surface.

**Figure 14:**
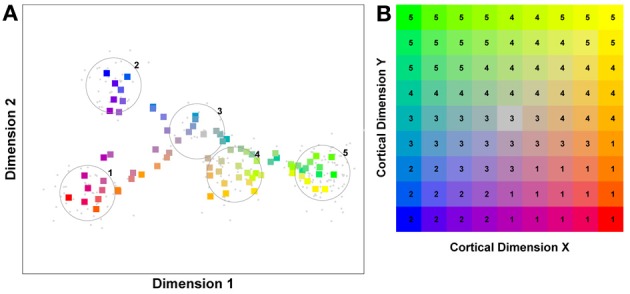
**Selectivity preferences for 81 neurons exposed to five object categories.** The classifiers were subject to strong local lateral excitation (*h* = 1), but otherwise the simulation parameters were identical to those shown in Figure 12. Notice in this case that there is evidence for quite rigid structure in neural selectivity, i.e., object category and physical location are now closely related. **(A)** Neural selectivity within object space. **(B)** Neural selectivity across the cortical surface.

The results of a new set of simulations appear in Figures 12–14. Within this space spanned by the two feature dimensions, five object categories were chosen on the assumption that exemplars from a category tend to cluster along the two input dimensions. Different numbers of exemplars in each category are generated, representing differing levels of exposure to the different categories (50, 40, 20, 50, and 100, respectively). Figure 12 shows the neural selectivity generated by the network when no lateral interaction is included. In the figure the color of the classifiers conveys their relative position in cortex, with similar colors corresponding to neighboring regions of cortex. In the absence of lateral interaction the neural classifiers are seen to distribute themselves randomly within and between the categories, suggesting no cortical clustering of neurons on the basis of input sensitivity/object category.

By contrast, the effect of even moderate, very local excitation is apparent in Figure 13. Now the structure of the cortical neighborhood is reflected in the distribution of neural selectivity preferences. Neurons tend to be selective for exemplars from the same object category as their immediate neighbors in cortex. Note, however, that there remain strong and sudden discontinuities in the patten of selectivity due to the clustering of objects in the input space. This pattern of locally smooth, yet punctuated stimulus preference, is broadly comparable to those described at the microscopic level in monkeys (Fujita et al., 1992; Tsunoda et al., 2001) and humans (Grill-Spector et al., 2006, 2007; Weiner and Grill-Spector, 2012), or at the broader level of faces vs. other objects (Zangenehpour and Chaudhuri, 2005; Kriegeskorte et al., 2008; Tsao et al., 2008b).

Including local excitation may have several important effects. In the lower layers of the network it may produce a local “association field”, of the type described by Field et al. (1993), and may lead to cells responding to the forms of illusory contours described in V2 neurons (Peterhans and von der Heydt, 1989; von der Heydt and Peterhans, 1989). In later layers it may also serve an important role in producing cells with similar response properties which has been shown to aid the learning of full view invariance (Tromans et al., 2012).

## Discussion

### Missing elements

The purpose of this paper has been to demonstrate how a self-organizing, competitive neural system can not only describe recognition in biologically inspired models of object recognition, but also in models of face recognition as well. The paper serves to unite earlier work on appearance-based models of face processing (Valentine, 1991; Valentine and Endo, 1992; Lewis and Johnston, 1999), with models of abstract-feature based models of face recognition (Bartlett et al., 2003; Jiang et al., 2006; Wallis et al., 2008) and biologically inspired models of object recognition (Fukushima, 1980; Wallis and Rolls, 1997; Riesenhuber and Poggio, 1999).

Nonetheless, as the title of the paper suggests, the models described here represent only a first step. The models do not attempt to explain tolerance to transformations and they ignore many details of what we know about early visual processing involving areas such as LOC (Eger et al., 2008). They also take early processing for granted. Thankfully there are excellent descriptions of how the simple feature analysers of early visual areas, such as “simple” and “complex” cell properties, can emerge in a self-organizing system (Hoyer and Hyvärinen, 2002).

At the other end of the processing hierarchy, the model does not explicitly model decision processes. This is a significant omission if one thinks that these higher areas almost certainly feed back into higher recognition areas, causing task-specific tuning of object-sensitive neurons (Wong et al., 2009b). There is also no attempt to explain the regional division of tasks and many regional specializations described in humans and primates (Wachsmuth et al., 1994; Haxby et al., 2000; Hoffman and Haxby, 2000; Meng et al., 2012). Like Tarr and Cheng (2003), I would argue that we have a core, self-organizing system that is picked over by multiple, task-oriented systems. This paper serves to explain how such a core system would operate, in terms of its adaptive encoding of objects of expertise, but not how these other systems come to extract information from it to solve specific tasks.

But why do we need multiple parallel systems you might ask? One important thing to bear in mind is that full view-invariance is only one possible goal of a visual system. Whether a person is facing toward you or looking at you provides a highly significant social cue which we care about, requiring us to retain object orientation information at some level too. Indeed, cellular recording provides ample evidence for neurons within the temporal lobe that are sensitive to head and eye gaze direction (Perrett et al., 1985; Hasselmo et al., 1989)—(see also Haxby et al., 2000). Needless to say, the importance of limiting view generalization extends to non-face objects too. In the special case of letter recognition it is important to know the difference between mirror and rotationally related letters such as “**d**,” “**b**,” “**p**,” and “**q**”. There is recent evidence that this may be the job of specific systems (Pegado et al., 2011). In a broader sense, it has been suggested that the diametrically opposing needs of systems aimed at answering “where” vs. “what” with respect to objects (e.g., Ungerleider and Haxby, 1994), are what drove the division of primate cortex into two separate streams (Wallis and Rolls, 1997). A fully integrated model of object and/or face recognition will have to understand these forms of regional specialization and multi-layer, multi-sensory integration.

The purpose of this summary is simply to point out that we might expect there to be numerous routes through the visual system and different termination points aimed at tapping into different multi-modal or view-specific sources of information (Bukach et al., 2006; Riesenhuber, 2007). Recent modeling papers from object recognition labs have also reflected this in their explanation for separate object and face recognition streams (Leibo et al., 2011). In practice this may place the wrong emphasis on what different streams are attempting to do. It may be the case that different aspects of face processing tap different functional streams. In other words, it may be the case that streams are divided more along functional than domain specific lines. As mentioned, some systems will be focussed on where a face is looking whereas others will be concerned with identification, for which viewing direction is irrelevant. The ability to extract these different types of object-specific information is presumably of interest when processing various aspects of non-face objects too. I would tend to agree with Riesenhuber and Poggio (2000) when they say that the different levels of representation required to solve specific tasks (view-independent, view-specific, categorization, identification) are all achievable through the action of the same underlying computational principles. What may affect the type of representation obtained in any one particular case will be a function of factors such as: where in the hierarchy the information is extracted, the degree to which temporal association of inputs is allowed to impact the representation, the extent of lateral excitation and/or inhibition, anatomical constraints, and the role of feedback from higher areas. What remains to be seen is whether such constraints are sufficient to explain the consistency as well as regularity of neural selectivity described in humans and primates (Kriegeskorte et al., 2008), which the model described here can, currently, only partially explain.

### Conclusion

The central message of this paper is that many phenomena related to face processing and the cortical arrangement of stimulus selectivity are all natural, emergent properties of a hierarchical, competitive, (abstract) feature-based face recognition system, a system which in essence, does not differ significantly from models describing human object recognition. The paper argues that faces are represented as pictorial features in much the same way as objects are. These features exhibit varying degrees of selectivity, transformation tolerance and extent, as a direct result of competitive processes within the visual processing stream. The precise response properties are a product of an individual's level of exposure to the relevant stimulus class. More exposure leads to greater numbers of neurons representing the stimuli with ever finer sensitivity to changes in appearance. Increasing the concentration of neural resources to a particular object class naturally produces more integrated and specialized selectivity and hence an ever more holistic representation. All of these phenomena emerge naturally from a self-organizing model sharing all of the fundamental elements of self-organizing models of object recognition. One can summarize the main messages of the paper as follows:

- To understand the sensitivity of neurons to objects and faces, one has to consider the behavior of a learning, self-organizing system.
- A system incorporating a hierarchy of competitive neural networks produces selectivity comparable to that known to exist in primate cortex.
- Including lateral excitation allows the system to produce spatial clustering of selection preferences similar to that described in humans and other primates.
- A self-organizing system does not divide the input space (of objects and faces) evenly. Neurons greedily cluster in areas of the space in which many exemplars exist. This leads to discontinuities in selectivity across the input space and the surface of temporal lobe cortex.
- Holistic representations emerge spontaneously in self-organizing competitive systems in regions of the input space where many exemplars are seen (i.e., in areas of visual expertise).
- Representations can be regarded as exemplar-based, abstract-features whose dimensionality/complexity/degree of input integration is driven by the proximity (in object space) of other neural classifiers.
- This proximity is determined by three factors: the regularity with which a stimulus in that region of input space is seen, the degree of physical similarity between exemplars, and the number of classifiers (neurons) active in that region of input space.
- An abstract-feature based system can explain adaptation after-effects and prototype effects if a final decision process is added on top of the feature-based/multi-channel representation (Wallis et al., 2008; Ross et al., 2013). This final processing layer would most likely lie in the frontal lobe, beyond the object recognition centers of temporal lobe cortex (Riesenhuber and Poggio, 2000).

As described above, there is plenty of debate and controversy relating to face processing and, specifically, the basis for holistic processing. Fundamental questions still exist relating to when, or indeed if, learning is required for holistic effects to emerge, and whether holistic effects map to other object classes, given sufficient exposure. Clearly my model would argue that they should. Evidence for the acquisition of holistic processing in the other-race effect would seem to point to the potential for holistic processing to be affected by experience. Whatever the link to the broader issue of expertise, the model offers a means for holistic face processing to emerge though learning in a system which bears the hallmarks of an accepted model of object recognition.

So what, if anything, can this add to the debate on the issue of whether face recognition is truly “special”, special in the sense that it is subserved by unique mechanisms (configural/holistic) and devoted neural hardware? The fact that the numerous behaviorally measured peculiarities of face recognition can be explained by a model which is also suitable for the recognition of objects, would seem to obviate the need for any specialist systems or pathways. In practice though, it is probably beyond the scope of this work to draw conclusions on that issue. What the model tells us is that despite the apparent peculiarity of responses to its preferred stimuli, the face recognition system can be viewed as a carbon copy of the object recognition system in terms of the associative and competitive mechanisms involved in its construction.

### Conflict of interest statement

The author declares that the research was conducted in the absence of any commercial or financial relationships that could be construed as a potential conflict of interest.

## Figures and Tables

**Figure A1:**
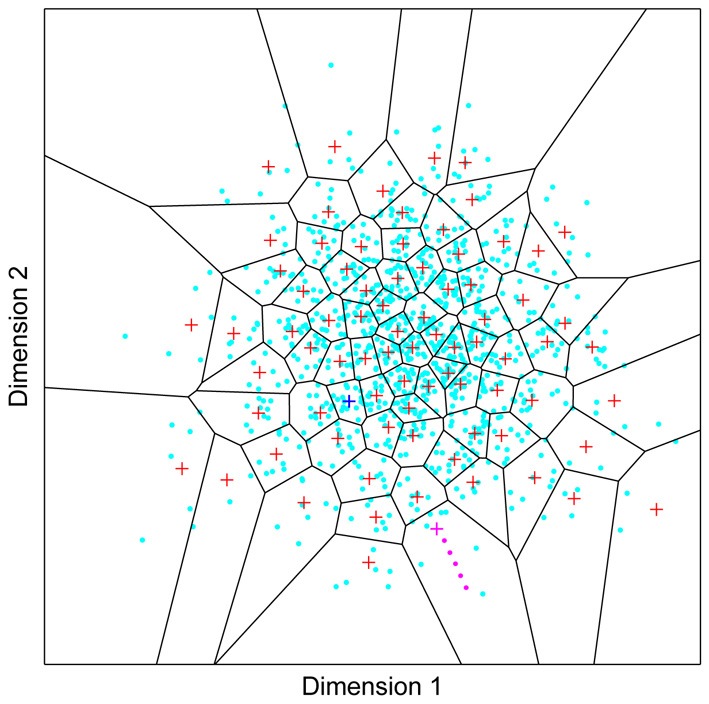
**Outcome of a self-organizing system exposed to 500 face images.** The light blue dots represent the projection of the 500 faces into two arbitrary dimensions of face space. If an individual’s face contains a feature which activates the classifier “+” highlighted in pink, then caricaturing (in this case increasing along Dimension 1 and decreasing along Dimension 2) will form an effective caricature. Caricaturing can be thought of as shifting the face along the trajectory of pink dots. Note that even at large distances from the point corresponding to the classifier’s weight vector (pink “+”), such images can be effective stimuli for the classifier because it experiences reduced inhibition from its neighboring neurons. If the face activates the classifier indicated in dark blue, neither dimension offers good scope for caricaturing. Hence a caricature artist would do well to choose an alternative dimension, of which there will be many.

## Footnotes

^1^Despite some early claims, the latest literature suggests that we share our sensitivity to inversion with monkeys (Phelps and Roberts, 1994; Perrett, 1988; Parr and Heintz, 2006; Tomonaga, 2007), although not all animals (Phelps and Roberts, 1994).

^2^Note that as a result of the projection, the true decision boundaries (shown in black in this and subsequent figures) are not straight, but are actually slightly curved. Hence the voronoi tessellation algorithm used to plot the boundaries is only approximately correct.

^3^Note that this assumes a winer-takes-all, single cell encoding scheme which is clearly a gross simplification. In practice, a biologically more relevant, distributed code would produce similar results. Such an approach is used later in the paper to analyze a more complex, high-dimensional model.

^4^Note that despite these promising links, holistic processing is probably only part of what drives the other-race effect, and outcomes have not always been consistent across all races (Mondloch et al., 2010; Crookes et al., 2013).

## Appendix

### Caricatures

As a final aside, I would just like to describe how models of the type described in this paper can also explain the “caricature effect”. As described in the main text above, this effect was seen as a challenge to exemplar-based models because even if a caricature has never been seen before, it often produces faster and more accurate recognition than an image of the real person it represents. Lewis and Johnston (1999) tackled this problem by considering decision space around an exemplar rather than its exact location in face space. The authors describe how the exact exemplar sits slightly offset from the centroid of the face decision boundaries in which it is active (this offset is usually directed approximately toward the grand mean of faces). The caricature, they argued, lies closer to the center of the decision boundary space. This captures the essence of why caricatures may be easier to recognize than the real face. Of course one may argue that faces near the center of the distribution of familiar faces do not have much room to move and so will show little or no caricature effect. Possibly, but it is worth adding that if one considers faces as being represented by a large number of dimensions, it need only be the case that the face is an outlier on one of these many dimensions for a caricature artist to be able to produce a compelling effect. As Ross et al. (2013) describe when considering high-dimensional representations of a face, “[For] faces to be clustered in the center of [all dimensions of a] multidimensional space... no face could ever have an extreme value along any of [the] several hundred dimensions. The likelihood of that ever happening is beyond remote.” A good caricature artist presumably seeks to highlight the feature or features which are already unusual (and hence outliers). A classifier associated with that feature should, therefore, occupy the edge of face space along that dimension and hence be likely to be strongly activated by any feature which occupies that region or many more peripheral points along that dimension.

In the context of a competitive network, the further from the grand mean a face lies the less the noise/clutter/competition a classifier experiences, and hence the greater its response. This point is illustrated in Figure A1 which represents the outcome of learning in a competitive system. The significant and counterintuitive point I wish to make is that in a competitive scheme, as an input exemplar moves away from the global mean it can produce greater response from its associated classifier because that classifier experiences greatly reduced competition from neighboring classifiers. In computational terms this means that a judiciously chosen input vector pointing away from the direction of the neural weight vector may nonetheless be a more effective stimulus for that neuron than one which exactly matches the neuron's weight vector.

Incidentally, papers that report the impact of adaptation on discrimination performance, describe how exposure to the grand mean of faces enhances discrimination performance across the population (Rhodes et al., 2010). Although possible to link such an effect to a norm-based model, it is apparent that effects of this type are also predicted by a multi-channel model as well. Adaptation effectively promotes the parts of each face that are outliers. One can think of the adaptation process as driving individuals to look more like their respective caricatures. The same basic augment to the one I am making in this section has been articulated in the past by Byatt and Rhodes (1998). In their paper the authors simultaneously manipulated the other-race and caricature effects in an attempt to tease apart norm-based and exemplar-based codes. They went on to conclude that an exemplar-based model best explained their results.

A recent set of data which also speaks to these types of effects was conducted on neurons in the middle-face patch of monkeys (Tsao et al., 2008a). In the study the authors reported how the systematic linear shifting of specific facial features of a cartoon face (e.g., inter-ocular distance, hair thickness, eyebrow angle etc.) caused a linear shift in neural response (if it produced any systematic change at all). In a separate paper, the lead author of that study argued that the results are consistent with a norm-based representation of faces (Tsao and Livingstone, 2008). In practice, shifting features around in this way may represent a crude form of caricaturing. If a neuron responds to the cartoon face it is likely that along one of the many feature dimensions being tested, one will prove to be a good feature for caricaturing. Hence responses will increase as the feature is exaggerated. When pushed in the opposite direction, the anti-caricature is formed and firing is driven below normal background firing rates—as is apparent in the data the authors report. One thing the data suggest is that the classifier boundaries are not hard cut-offs, a neuron does not cease to fire at the point the input matches the favored stimulus of a neighboring neuron along that dimension of input space. That need not be surprising as the stimulus remains an effective stimulus for that neuron along all other feature dimensions (e.g., nose width, eye color etc.) and which are not currently being altered.
